# A yeast synthetic biotic platform for delivery of therapeutic nanobodies to ameliorate gastrointestinal inflammation

**DOI:** 10.1242/dmm.052620

**Published:** 2026-03-09

**Authors:** Roger Palou, Almer M. van der Sloot, Aline A. Fiebig, Megan T. Zangara, Naseer Sangwan, María Sánchez-Osuna, Bushra Ilyas, Haley Zubyk, Michael Cook, Gerard D. Wright, Brian K. Coombes, Mike Tyers

**Affiliations:** ^1^ Program in Molecular Medicine, The Hospital for Sick Children Research Institute, Toronto, ON M5G 0A4, Canada; ^2^ Mila - Québec Artificial Intelligence Institute, Montréal, QC H2S 3H1, Canada; ^3^ Université de Montréal, Département d'informatique et de recherche opérationnelle, Montréal, QC H3C 3J7, Canada; ^4^ Department of Biochemistry and Biomedical Sciences, McMaster University, Hamilton, ON L8S 4K1, Canada; ^5^ Microbial Sequencing and Analytics Resource (MSAAR) Facility, Lerner Research Institute, Cleveland Clinic, Cleveland, OH 44106, USA; ^6^ Center for Microbiome and Human Health, Cleveland Clinic, Cleveland, OH 44106, USA; ^7^ David Braley Center for Antibiotic Discovery, Michael G. DeGroote Institute for Infectious Disease Research, McMaster University, Hamilton, ON L8S 4K1, Canada; ^8^ Department of Molecular Genetics, University of Toronto, Toronto, ON M5S 1A8, Canada

**Keywords:** *Saccharomyces boulardii*, Synthetic biotic, VHH nanobody, Inflammatory bowel disease, TNF

## Abstract

Protein-based pharmaceuticals, such as engineered antibodies, form a major drug class of steadily increasing market share. However, these biologic medicines are costly to manufacture, are subject to strict supply chain and storage constraints, and often require invasive administration routes. Engineered microbes that secrete bioactive products directly within the microbiome milieu may mitigate these challenges. Here, we describe a cell microfactory platform based on the probiotic yeast *Saccharomyces boulardii* for the production of nanobody biologics in the gastrointestinal (GI) tract. High-level secretion of nanobodies by *S. boulardii* was achieved by optimizing promoters, secretion signals and antibody formats. In mice, oral gavage of *S. boulardii* allowed efficient and transient colonization of the colonic compartment, and *in situ* production of a therapeutic nanobody directed against tumor necrosis factor (TNF). In a mouse model of chemical-induced colitis, GI-delivery of anti-murine TNF nanobody via live *S. boulardii* improved both survival and disease severity without causing overt perturbation of microbiome composition. These results position *S. boulardii* as a synthetic biotic platform for the *in situ* production and delivery of protein-based therapeutics to the GI tract.

## Teaser

**Editor's choice:** *Saccharomyces boulardii* is developed as a synthetic biotic platform for *in situ* production of protein-based therapeutics in the gastrointestinal tract. *S. boulardii* engineered to secrete a neutralizing nanobody against tumor necrosis factor improved outcomes in a mouse model of colitis.

## INTRODUCTION

The concept of using live microbial cells to manufacture and deliver therapeutic agents to the microbiome – variously termed synthetic biotics, engineered probiotics, cell microfactories or living therapeutics – holds promise for cost-effective, site-specific therapeutic interventions (Chua et al., 2017; Heavey et al., 2022). Synthetic biotics may be designed to produce biosynthesized small molecules – such as nutrients, vitamins and antibiotics – or more complex protein-based agents – such as antibodies, peptides, growth factors and cytokines. The *in situ* synthesis of active biomolecules in the gastrointestinal (GI) tract by cell microfactories is now well-established for bacterial platforms, including *Lactobacillus lactis*, *Lactococcus lactis* and *Escherichia coli* strain Nissle 1917 (Lynch et al., 2023; Mimee et al., 2016; Vandenbroucke et al., 2010; Wu et al., 2024). However, the use of bacterial species as orally administered synthetic biotics can be compromised by loss of viability in the acidic environment of the stomach, the inability to produce high levels of secreted complex biologic molecules, such as antibodies, and the risk of lateral gene transfer to other species in the microbiome or external environment (Mimee et al., 2016; Munck et al., 2020). Although the gut microbiome is dominated by bacterial species, fungal, archaeal and protozoal species form another important component of the microbiome (Richard and Sokol, 2019).

To overcome the problems inherent to bacterial-based synthetic biotics, the yeast *Saccharomyces boulardii* has been explored as an alternative platform for *in situ* production of biologic agents (Chen et al., 2020; Deleu et al., 2024; Durmusoglu et al., 2021; Liu et al., 2020). *S. boulardii* is closely related to the budding yeast *Saccharomyces cerevisiae* (Khatri et al., 2017), also known as baker's or brewer's yeast, a mainstay model organism for eukaryotic molecular genetics that is also widely used in food and biotechnology production processes. *S. boulardii* is a ‘generally recognized as safe’ organism that is widely used as an over-the-counter probiotic for the treatment of *Clostridium difficile* infections and antibiotic-associated diarrhea (Kelesidis and Pothoulakis, 2012). Moreover, in pre-clinical studies, *S. boulardii* has been shown to reduce inflammation in a mouse colitis model (Dalmasso et al., 2006). *S. boulardii* has inherent potential advantages as a synthetic biotic, including relative ease of genetic manipulation that benefits from extensive molecular genetic tools developed over many decades for *S. cerevisiae*. Like budding yeast, *S. boulardii* can be readily produced at an industrial scale and at low cost, retains long-term viability in desiccated form and shows negligible rates of lateral gene transfer (Kelesidis and Pothoulakis, 2012; Sen and Mansell, 2020). Moreover, *S. cerevisiae* spp. are part of the normal human mycobiome (Hudson et al., 2016). Unlike *S. cerevisiae*, however, *S. boulardii* exhibits increased acid tolerance, elevated resistance to gastric and intestinal fluids, and pronounced immunomodulatory activity (Duffey et al., 2025 preprint; Hudson et al., 2016). These physiological properties make *S. boulardii* well suited for development as a synthetic biotic platform in humans.

Previous work has demonstrated the biosynthesis of small molecules (e.g. β-carotene, violacin) and recombinant proteins (e.g. HIV-GAG, lysozyme, IL-10, anti-*C. difficile* nanobody tetramers) using engineered *S. boulardii* strains (Chen et al., 2020; Durmusoglu et al., 2021; Li et al., 2021; Liu et al., 2016, 2020; Palma et al., 2019; Rebeck et al., 2025). Currently, biopharmaceuticals or biologics and, in particular, recombinant monoclonal antibodies (mAbs) or other antibody-like molecules, comprise more than 20% of new drug approvals and offer important new therapies for a variety of autoimmune diseases and cancers (Mullard, 2021). Notwithstanding these successes, antibody-based therapeutics present major challenges for therapeutic use, including expensive manufacturing and quality control, product instability, cold chain requirements for storage and distribution, pharmacokinetic limitations, and injection-based delivery. These liabilities preclude access for many patient populations and impose substantial costs to healthcare systems.

Inflammatory bowel disease (IBD), including Crohn's disease (CD) and ulcerative colitis (UC), are chronic inflammatory disorders of the GI tract caused by a combination of host genetics, environmental factors and microbiome composition (Wlodarska et al., 2015). CD and UC are debilitating, life-long conditions that reduce quality of life and often result in complications that require surgery or other interventions. Tumor necrosis factor (TNF, also known as TNFα) and other inflammatory cytokines are primary effectors of bowel inflammation and contribute to microbiome dysbiosis in CD (Wlodarska et al., 2015). A validated therapeutic approach is the administration of anti-TNF mAbs to suppress the immune response (Kim, 2021). Although successful in achieving remission for some CD patients, treatment with an injectable mAb is cumbersome and expensive at more than $10,000 per patient per year (Burisch et al., 2025). Unfortunately, mAbs against TNF have shortcomings that include failure to provide durable relief, unpredictable pharmacodynamics, systemic immune suppression, immunogenicity and complex dosing regimens that preclude consistent attainment of therapeutically effective levels (Vulliemoz et al., 2020). For these reasons, we focused on the development of *S. boulardii* as a synthetic biotic for the *in situ* production of neutralizing anti-TNF antibodies in the GI tract.

**Fig. 1.**
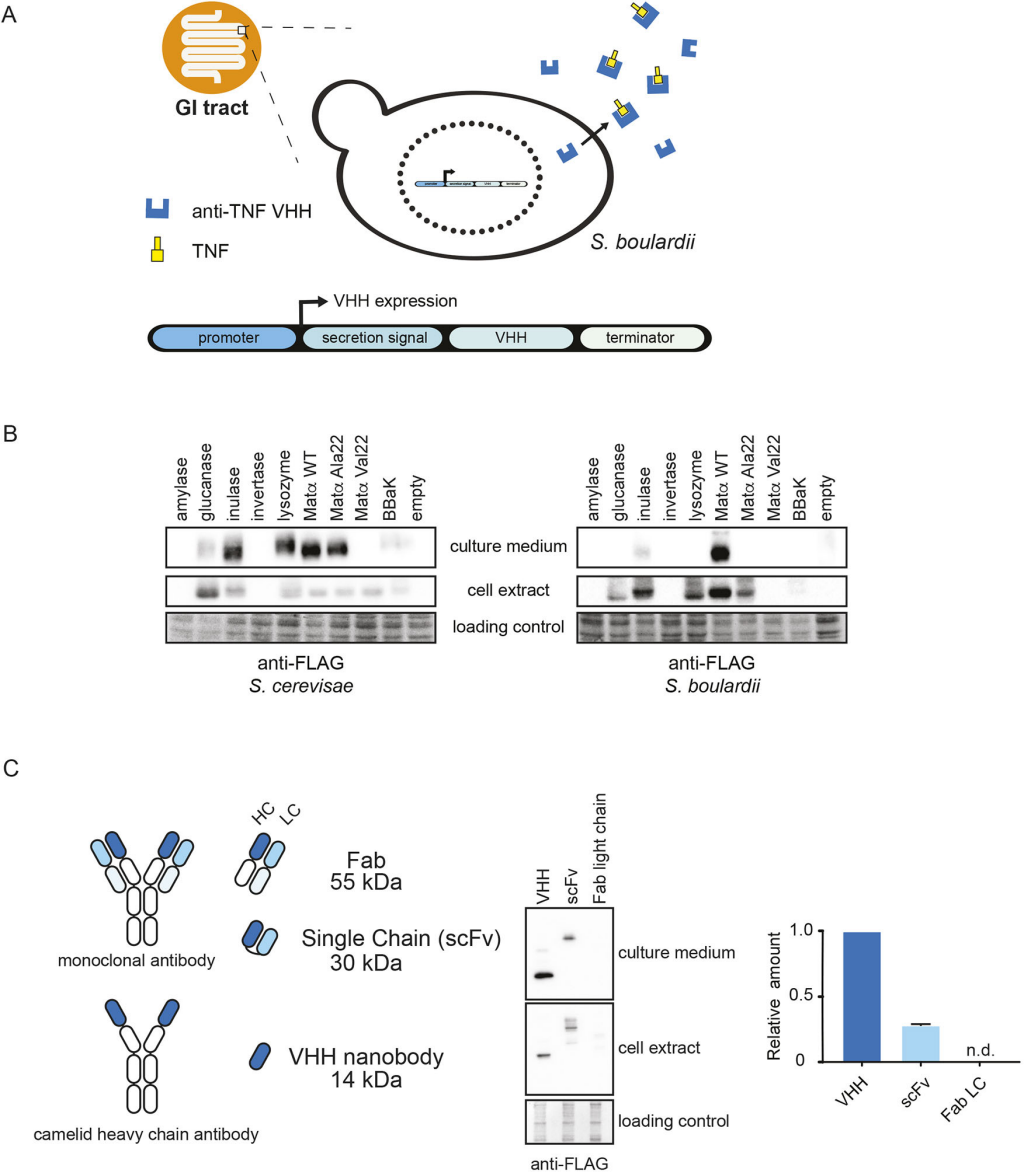
**Optimization of secretion signals and antibody scaffold formats.** (A) Overview of *S. boulardii* synthetic biotic platform. (B) Immunoblot to evaluate secretion signal efficiency. *S. cerevisiae* (left) and *S. boulardii* (right) were transformed with a galactose-inducible plasmid containing the light chain of a Fab′ against TcdA fused to nine secretion signals at the N-terminus and the FLAG epitope at the C-terminus. Cells were grown in SC-Ura medium with 1% galactose+1% raffinose at 30°C for 3 days in a shaker-plate reader. Secreted (culture medium) and retained (cell extract) Fab′ were detected by anti-FLAG immunoblot. Ponceau S stain was used as a loading control for cell extracts. (C) Immunoblot to evaluate antibody formats. S*. boulardii* was transformed with expression plasmids for a nanobody (VHH) against HIV-1 spike protein, a single-chain antibody (scFv) against TcdA or a light chain Fab′ fragment against TcdA, all fused to the Matα wild-type secretion signal (left). Cells were grown in SC-Ura medium with 1% galactose+1% raffinose at 30°C for 3 days. Retained and secreted Fab′ were detected by anti-FLAG immunoblot (middle). FLAG signals were quantified and normalized to VHH (right). Means±s.d. are indicated. n.d., not detected.

We optimized gene expression, protein secretion signals and antibody-like scaffolds to build a robust *S. boulardii* synthetic biotic platform to produce biologics in the GI tract (Fig. 1A). Nanobodies (also known as single-domain antibodies) based on the camelid variable heavy-chain domain (VHH) scaffold yielded far higher levels of secretion than conventional antibody variants (Steeland et al., 2016). We optimized the formulation and oral administration of *S. boulardii* for VHH delivery to the GI tract in mouse. Administration of *S. boulardii* that secretes anti-TNF VHH to mice resulted in transient high-level colonization of the GI tract and *in situ* production of VHH at therapeutic concentrations without altering overall microbiome composition. An anti-TNF VHH-secreting strain significantly improved survival compared to control *S. boulardii* strains in a Gehan–Breslow–Wilcoxon (DSS)-induced colitis mouse model. These results demonstrate that localized production and delivery of a therapeutic VHH nanobody via *S. boulardii* mitigates inflammatory disease activity in the gut.

## RESULTS

### Secretion tags and antibody scaffolds

Protein secretion requires the presence of a signal peptide at the N-terminus of the protein of interest to engage with the secretory pathway (Hou et al., 2012). To enable the secretion of heterologous antibody scaffolds, nine candidate secretion signal peptide sequences were fused to the N-terminus of the light chain of an engineered fragment antigen-binding (Fab′) dimer directed against *C. difficile* toxin A (TcdA) (Wong, 2013) and tested for secretion yield. Secretion signals were derived from *S. cerevisiae* (i.e. amylase, glucanase, inulase, invertase), chicken (lysozyme), three variants of the *S. cerevisiae* mating factor alpha (Matα) leader peptide [i.e. wild type; Matα variant App8 (Ala22a) and variant Val22 (Val22a) (Rakestraw et al., 2009)], and a synthetic consensus signal peptide called BBaK (Clements et al., 1991) (see Table S1 for all sequences and sources). These signal peptides have previously been shown to support protein secretion of antibodies, antibody-like scaffolds and other heterologous proteins in *S. cerevisiae* (Li et al., 2002). Signal sequence Fab′ constructs were expressed under the control of the *GAL1*-inducible promoter on a 2-μm high-copy *URA3* plasmid to alleviate potential toxic effects of Fab′ expression and/or secretion on yeast growth. As wild-type *S. boulardii* yeast lacks the common auxotrophic markers available in *S. cerevisiae* laboratory strains (Khatri et al., 2017), an auxotrophic *S. boulardii* strain was engineered using CRISPR-Cas9-mediated knockout of *URA3*, to facilitate cloning and allow plasmid interoperability between *S. cerevisiae* and *S. boulardii*. To allow CRISPR-based gene engineering, we generated a plasmid that encodes *S. pyogenes* Cas9 and the *Aspergillus nidulans* gene acetamidase (*amdS*), a dominant prototrophic marker that allows *S. cerevisiae* to grow on acetamide as the sole nitrogen source (Solis-Escalante et al., 2013). After gene editing, removal of the Cas9 plasmid was achieved by counter selection on fluoroacetamide, which generates a toxic metabolite (Solis-Escalante et al., 2013). This method allowed scarless gene editing and subsequent elimination of the vector containing Cas9 and the sgRNA to minimize possible off-target cutting effects (Liu et al., 2016). The signal-peptide constructs were first tested by using a Fab′ fragment in the wild-type *S. cerevisiae* strain Sigma1278b. All signal peptides enabled secretion of the Fab′ fragment, except the amylase, invertase and Matα Val22 constructs (Fig. 1B, left). The Matα wild-type sequence was the most-efficient secretion signal in *S. cerevisiae*, with only low amounts of detectable Fab′ fragments remaining inside the cell. In *S. boulardii*, only the Fab′ fragment fused to a Matα wild-type signal peptide or, to a lesser extent, the inulase signal peptide, was expressed and secreted. We also observed higher retention of the Fab′ inside *S. boulardii* cells compared to the same constructs in *S. cerevisiae* cells (Fig. 1B, right). As the Matα leader sequence is conserved between *S. boulardii* and *S. cerevisiae* (Khatri et al., 2017) the basis for this difference is unclear. These results illustrate that even highly related yeast species can exhibit unexpected differences, underscoring the need for systematic characterization of chassis parts in biotechnological applications.

### Optimization of antibody scaffolds

Without considerable additional engineering of the secretion system, yeast species such as *S. cerevisiae* and *Pichia pastoris* are relatively inefficient in expressing canonical full-length antibodies (de Ruijter et al., 2016). To overcome the requirement for Fab′ light-heavy chain heterodimer expression, we assessed the production of human antibody-derived single-chain variable fragments (scFv) and monomeric antigen-binding VHH domains derived from camelid species, referred to as nanobodies. Both antibody-like scaffolds (Fig. 1C) have favorable properties compared to conventional antibodies or Fab fragments, including smaller size and improved protein stability, while maintaining similar affinity for the target as conventional antibodies (Steeland et al., 2016). To determine which antibody scaffold format was most compatible with *S. boulardii*, we compared the expression and secretion of a scFv against *Staphylococcal* enterotoxin B (scFv-GC132) (Chen et al., 2019), a Fab against *C. difficile* toxin A (ToxA-A03) (Wong, 2013) and a VHH against HIV-1 spike protein (McCoy et al., 2012) (Fig. 1C, left). Scaffolds were fused to the Matα wild-type secretion-signal peptide and expressed under the control of *GAL1* promoter. In *S. boulardii*, the expression and secretion of the VHH construct was four times higher than the scFv construct, while the Fab light chain was not detectably expressed (Fig. 1C, middle and right). The VHH format was, therefore, chosen as the preferred scaffold for further studies.

### Optimization of VHH production

**Fig. 2.**
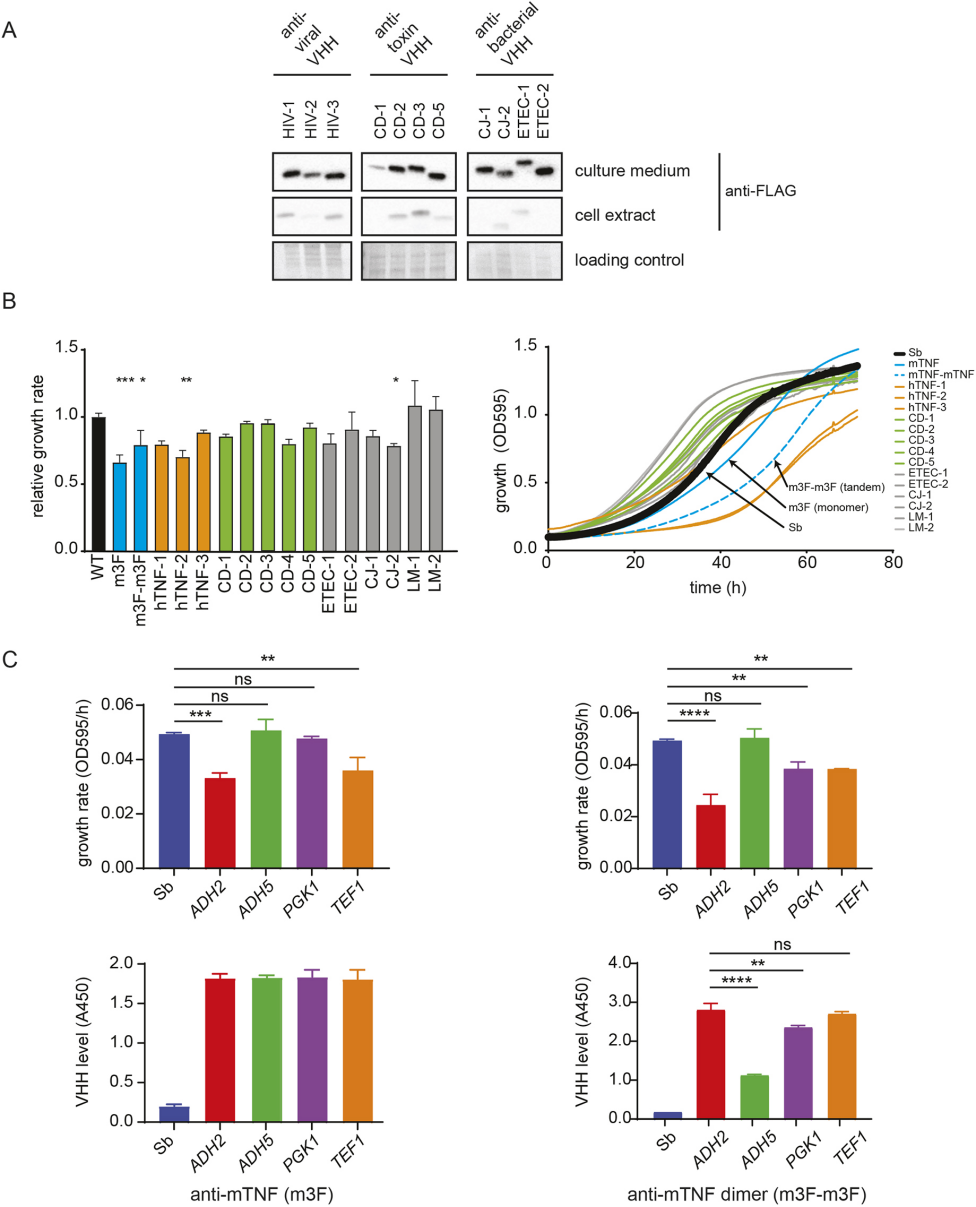
**Characterization of secreted VHH nanobodies in *S. boulardii*.** (A) Western blot showing VHH nanobody secretion in *S. boulardii.* A panel of VHHs against HIV gp140 (HIV-1, HIV-2, HIV-3), *Clostridium difficile* TcdA (CD-1, CD-2, CD-3) and TcdB (CD-5) toxins and enterotoxigenic *E. coli* (ETEC) F4 fimbrae (CJ-1, CJ-2) and Stxe2 (ETEC-1, ETEC-2) were FLAG tagged and constitutively expressed from the ADH2 promoter in *S. boulardii* grown at 37°C for 3 days in a shaker-plate reader. Retained and secreted VHH from 60 µl of culture were detected by anti-FLAG immunoblot. Ponceau S stain was used as a loading control for cell extracts. (B) Effect of VHH secretion on *S. boulardii* growth rate. A panel of the indicated VHHs directed against various antigens were N-terminally fused to the Matα wild-type secretion signal and expressed from the *ADH2* promoter. Growth in liquid culture was monitored by OD_595_ and normalized to wild-type growth rate based on curve slope during exponential phase. Statistical analysis was performed using Dunnett's ANOVA test comparing each strain to wild type (*P<0.05, **P<0.01, ***P<0.001). Means±s.d. of three independent experiments are indicated. (C) Optimization of anti-mouse TNF (mTNF) VHH expression *in vitro*. Anti-mTNF VHH (m3F) was expressed from the indicated yeast promoters. Top row: growth rates determined from growth curves. Bottom row: secreted mTNF VHH levels quantified by ELISA. Statistical analysis was performed using Tukey's ANOVA test (**P<0.01, ***P<0.001, ****P<0.0001; ns, not significant). Means±s.d. of three independent experiments are indicated.

Use of *S. boulardii* as a living cell microfactory platform to deliver protein-based therapeutic agents to the GI tract requires high-level expression and secretion of the protein of interest. Promoters used to drive expression should ideally be active in the low-oxygen and low-glucose environment of the distal GI tract (Albenberg et al., 2014). We considered the *ADH2* promoter (*ADH2^pr^*) as a potential constitutive promoter candidate, as it is highly active at low glucose concentrations and under hypoxic conditions (Lai et al., 2006). To assess the potential influence of protein sequence on expression, a panel of VHHs with different sequence characteristics was cloned into a 2-µm plasmid under the control of *ADH2^pr^* and N-terminally fused to the Matα wild-type signal peptide (Lai et al., 2006). For these experiments, we used the prototrophic *S. boulardii* parental strain (MYA-796), rather than a *ura3* auxotrophic derivative, to maximize strain fitness. We expressed various *ADH2^pr^-VHH* constructs, by using a 2-µm plasmid bearing *kan* antibiotic selection that confers resistance to G418. This selection marker had the added benefit of allowing recovery and assessment of VHH-expressing *S. boulardii* strains after passage through the mouse GI tract (see below). *S. boulardii* strains bearing these plasmids expressed, secreted and properly processed the Matα-VHH fusion proteins at similar levels, such that – on average – 95% of each VHH was secreted (Fig. 2A).

A reduction in fitness of engineered *S. boulardii* strains could cause a deleterious effect on growth *in vivo* and represent a potential liability for colonization of the GI tract, especially in competition with other *Saccharomyces* species in the host mycobiome (Yan et al., 2024). To analyze the impact of VHH expression and secretion on *S. boulardii* fitness, we assessed yeast growth at human physiological temperature (37°C) and used growth rate as a proxy for fitness. Constitutive expression of VHHs did not impact *S. boulardii* growth rate for 12 out of 16 different VHH nanobodies tested (Fig. 2B; Fig. S1). Unfortunately, the VHHs that adversely affected fitness included an anti-mouse TNF (mTNF) VHH (m3F) and a corresponding tandem VHH dimer (m3F-m3F), which we had prioritized for use in *in vivo* experiments (see below). For these constructs, we explored whether the promoter controlling VHH expression could have an impact on cell fitness. The growth rate of strains expressing either the m3F anti-mTNF VHH or the m3F-m3F VHH dimer, each under the control of four different promoters (*ADH2*, *ADH5*, *PGK1* and *TEF1*) (Lai et al., 2006), was compared during 3 days of growth at 37°C. ELISA was used to measure the amount of secreted VHH monomer or tandem VHH dimer after 3 days. For strains expressing the VHH monomer, the *ADH5* and *PGK1* promoters fully rescued cell fitness without compromising VHH secretion. In contrast, although the use of the *ADH5* promoter rescued fitness for the VHH dimer strain, this was accompanied by a substantially decreased yield of secreted VHH dimer (Fig. 2C). These optimization steps yielded an *S. boulardii* strain that optimally expressed an anti-mTNF VHH monomer at 37°C without overt fitness defects.

### Optimization of VHH secretion

Deletion of the *HDA2*, *VPS5* and *TDA3* genes – all three of which are implicated in trafficking and secretory functions – can increase protein secretion in *S. cerevisiae* (Huang et al., 2018). The Cas9-*amdS* counter selection plasmid was used to generate CRISPR-mediated deletions for these three genes in *S. boulardii*. We analyzed the secretion of the anti-mTNF VHH under the control of the *ADH2*, *ADH5*, *PGK1*, *TEF1* or *TDH3* promoters. Secretion of each VHH was increased at 30°C in the individual knockout strains, except when expression was controlled by the *ADH2* promoter. At 37°C, the triple *hda2D vps5D tda3D* mutant expressing the anti-mTNF VHH under control of the *PGK1* promoter exhibited the highest level of VHH secretion, ∼1.4-fold more than in the corresponding wild-type strain (Fig. S2B). However, all single, double and triple deletion mutants adversely affected cell growth rate at both 30°C and 37°C (Fig. S2B,C). Owing to this evident growth disadvantage compared to wild-type *S. boulardii* and the relatively minor improvement in VHH secretion yield, these gene deletions were not included in the final *S. boulardii* chassis used for VHH delivery *in vivo*.

### Secretion of functional anti-TNF VHH

**Fig. 3.**
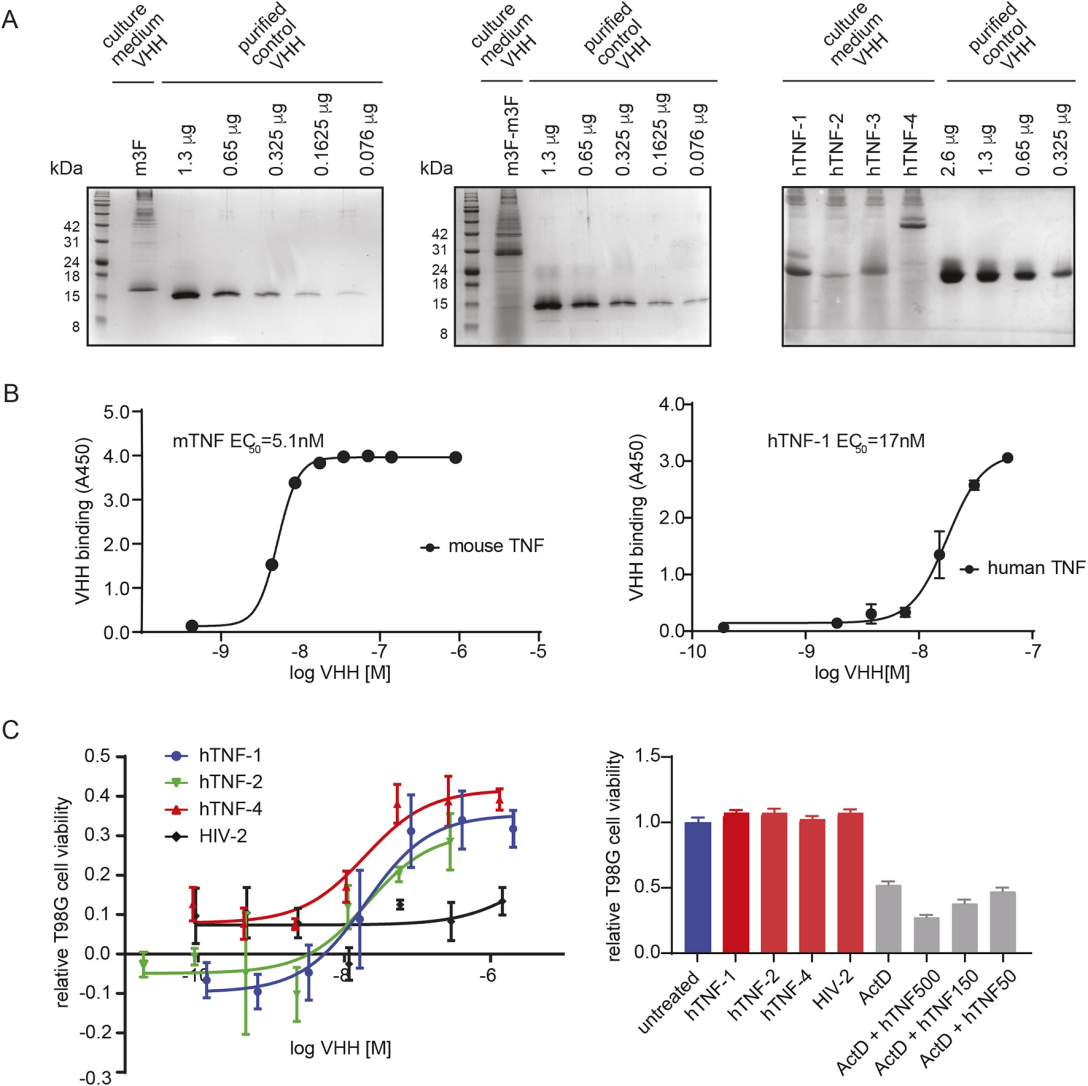
**Characterization of anti-TNF VHH nanobodies produced in *S. boulardii*.** (A) SDS-PAGE showing secreted levels of indicated VHH in yeast culture medium (60 µl), quantified by comparison to a dilution series of purified recombinant His-tagged control nanobody (CD-2). Proteins were detected with Coomassie Brilliant Blue stain. Left: secreted anti-mTNF m3F VHH. Middle: *s*ecreted anti-mTNF m3F-m3F VHH tandem dimer. Right: secreted anti-hTNF VHH (hTNF-1, hTNF-2, hTNF-3) and anti-hTNF VHH tandem dimer (hTNF-4=hTNF-3/hTNF-1). Estimated concentrations in culture medium were: 6.9 µg/ml for anti-mTNF m3F VHH; 4.4 µg/m for anti-mTNF m3F-m3F tandem dimer VHH; 5.5 µg/ml for hTNF-1 VHH; 1 µg/ml for hTNF-2 VHH; 3.4 µg/ml for hTNF-3 VHH; 4.9 µg/ml for hTNF-4 tandem VHH. (B) Binding affinities of secreted anti-TNF VHH nanobodies against mouse TNF (mTNF; left) and human TNF (hTNF; right). Binding of anti-mTNF VHH (m3F) to mTNF or anti-hTNF hTNF-1 VHH to hTNF was quantified by ELISA. Means±s.d. are indicated. (C) Neutralization of hTNF-induced cytotoxicity in human T98G glioblastoma cells treated with 40 nM actinomycin D to sensitize cells to hTNF. Left: effect of indicated anti-hTNF VHHs on viability of cells treated with 500 ng/ml hTNF in the presence of 40 nM actinomycin D. An anti-HIV-1 VHH (HIV-2) served as a non-specific VHH control. Means±s.d. are indicated. Right: cell viability 48 h after treatment with the highest concentrations of anti-hTNF VHHs used in neutralization assays (2.04 µM for hTNF-1; 0.28 µM for hTNF-2; 1.33 µM for hTNF-4; 1.45 µM for HIV-2). Viability effects of 40 nM actinomycin D alone or in the presence of the indicated concentrations of hTNF are also shown. Means±s.d. are indicated.

We then quantified the yield of the secreted anti-TNF VHHs and tested for binding and neutralizing activity *in vitro*. We extended this analysis to human TNF (hereafter referred to as hTNF) in anticipation of future use of *S. boulardii*-based synthetic biotics in humans. VHHs directed against mTNF (m3F) and hTNF (hTNF-1, hTNF-2 and hTNF-3) were all produced and secreted (Fig. 3A). The maximum concentration in the culture supernatant was 6.9 µg/ml for the hTNF-1 VHH, a yield that is sixfold higher than previously reported for an anti-hTNF VHH secreted by the Gram-positive bacterial probiotic *L. lactis* (Vandenbroucke et al., 2010). VHH oligomerization can increase apparent binding affinity to its target (Steeland et al., 2016), for example an anti-hTNF-3–anti-hTNF-1 VHH tandem dimer (hTNF-4) has 1000 times more affinity for hTNF than the corresponding VHH domain alone (Beirnaert et al., 2017). Despite the VHH dimer being similar in size to a scFv scaffold, *S. boulardii* secreted the VHH dimers at similar concentrations to the single VHHs, for both the hTNF-4 and m3F-m3F dimers (Fig. 3A). We used ELISA to demonstrate that the various secreted anti-TNF VHHs can bind recombinant TNF. The VHHs secreted by *S. boulardii* were functional and had a similar affinity (EC_50_) towards recombinant mouse and human TNF (Fig. 3B), as previously reported for *E. coli*-produced VHH (Beirnaert et al., 2017; Coppieters et al., 2006).

As a biological readout for the inhibition of hTNF by anti-hTNF VHH, we measured cell viability in the T98G human glioblastoma cell line, in which actinomycin D was used to sensitize the cells to hTNF (Sakuma et al., 1993; Wong et al., 1989). Anti-hTNF VHHs secreted by *S. boulardii* were able to protect against hTNF-induced cytotoxicity with high potency (EC_50_<100 nM), whereas an irrelevant control VHH had no effect (Fig. 3C, left). Importantly, we did not observe non-specific toxicity when T98G cells were treated with VHHs alone (Fig. 3C, right). These results demonstrated that anti-hTNF VHHs secreted by *S. boulardii* retained neutralizing activity *in vitro*.

### *S. boulardii* colonization is influenced by the endogenous murine microbiome, pH and dose

**Fig. 4.**
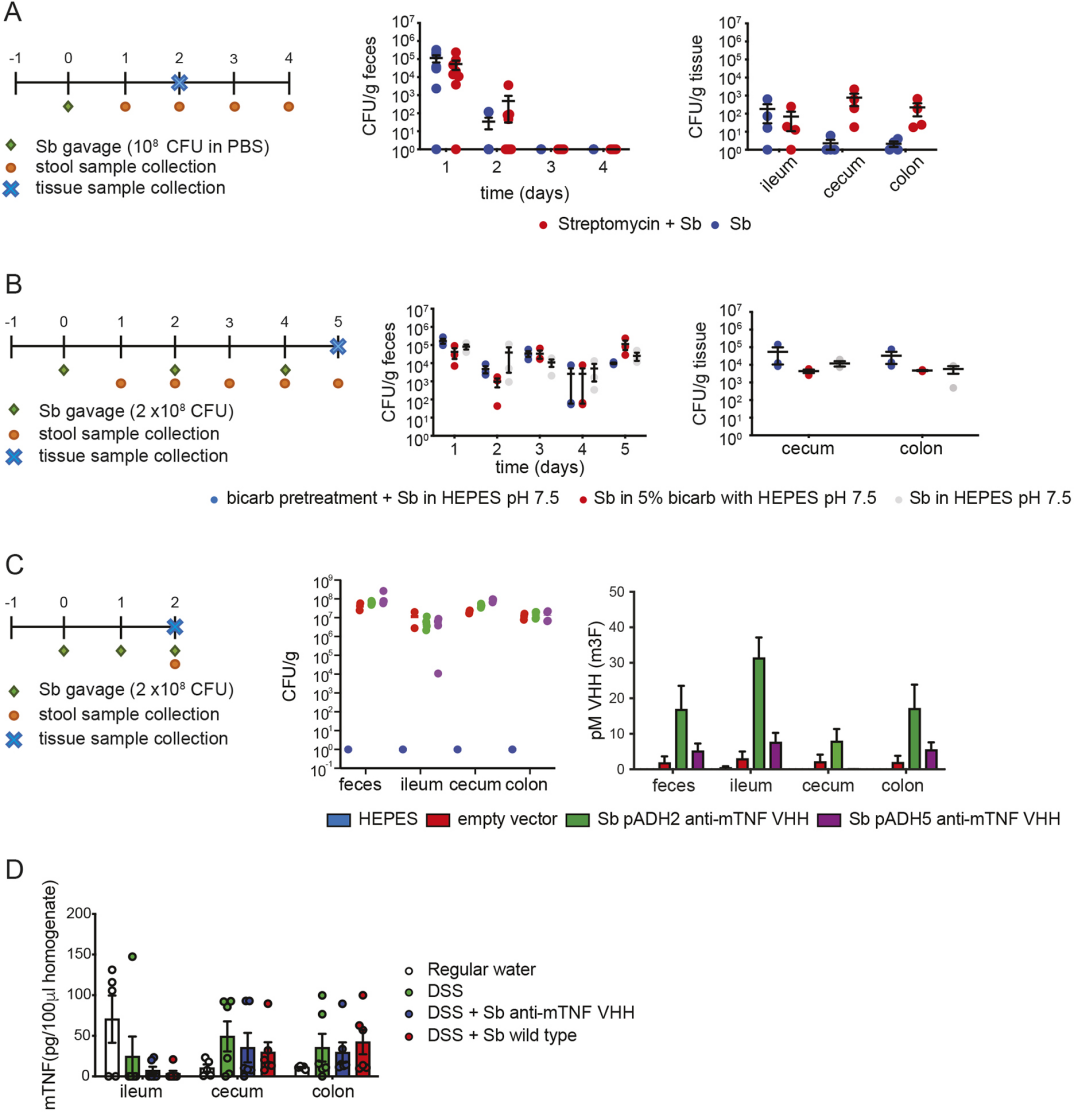
***In situ* production of anti-mTNF in the mouse GI tract by *S. boulardii*.** (A) Colonization of the GI tract by *S. boulardii*. Animals were untreated or pretreated with a single oral dose of 20 mg streptomycin 24 h prior to gavage with a single dose of 10^8^ CFU of wild-type *S. boulardii* in PBS bearing an empty vector (left). Quantification of CFU/g in feces by serial dilution after 4 days (middle) and in the indicated tissues after 2 days (right). (B) Effect of increased dose and pH neutralization on *S. boulardii* colonization. Animals were pretreated with a single oral dose of 20 mg streptomycin 24 h prior to treatment onset and then gavaged on days 0, 2 and 4 with 2×10^8^ CFU *S. boulardii* bearing an empty plasmid. Animals were either pretreated with 5% NaHCO_3_ prior to gavage in HEPES-buffered saline (0.9% NaCl, 100 mM HEPES pH 7.5), or gavaged with 5% NaHCO_3_+HEPES-buffered saline, or gavaged with HEPES-buffered saline only (left). CFUs were determined by serial dilution as in B (middle, right). Means±s.d. are indicated. (C) Quantification of anti-mTNF VHH production from two different promoters in the GI tract. Animals were pretreated with a single oral dose of 20 mg streptomycin 24 h then gavaged on days 0, 1 and 2 with 2×10^8^ CFU *S. boulardii* transformed with an empty plasmid or plasmids expressing anti-mTNF VHH under the control of the *ADH2* or *ADH5* promoter (left). *S. boulardii* CFU per gram of feces or tissue after 3 days of treatment under the HEPES-buffered saline regime for indicated strains were determined (middle). Anti-mTNF VHH was quantified in feces and tissues by ELISA for indicated strains after 3 days of treatment (right). Purified anti-mTNF VHH (m3F) produced in *E.coli* was used to generate a standard curve for ELISA. (D) Quantification of mTNF levels in tissues by ELISA.

Previous studies have suggested that *S. boulardii* does not stably colonize the murine GI tract in the absence of inflammation (Hudson et al., 2016). One possible explanation for this observation is that *S. boulardii* is partially outcompeted by the endogenous microbiome. To explore this possibility, C57BL/6 mice were treated with a single dose of streptomycin for 24 h, which is known to reduce colonization resistance of the mouse gut (Stecher et al., 2005). Mice were then gavaged with a single dose of 2×10^8^ colony-forming units (CFUs) of *S. boulardii* resuspended in phosphate-buffered saline (PBS; 10 mM disodium hydrogen phosphate pH 7.4, 137 mM NaCl, 2.7 mM KCl) and the residence time with or without streptomycin was determined by plating serial dilutions of resuspended feces on G418 medium to select for the *kan* resistance marker plasmid. In both streptomycin-pre-treated and control mice, *S. boulardii* CFUs recovered from feces peaked 24 h post gavage, and were undetectable after 72 h (Fig. 4A, middle). However, when we examined the colonic tissue population of *S. boulardii* 2 days after gavage, mice pre-treated with streptomycin had almost two orders of magnitude higher *S. boulardii* colonization in the cecum and colon compared to untreated controls (Fig. 4A, right).

Despite better survival of *S. boulardii* over *S. cerevisiae* under acidic conditions (Hudson et al., 2016), the low pH of the stomach might still affect *S. boulardii* fitness and decrease colonization rates. To test whether delivery under more alkaline conditions might improve colonization, mice were pre-treated with a 5% bicarbonate-buffered solution followed by gavage with wild-type *S. boulardii* in HEPES-buffered saline (0.9% saline, 100 mM HEPES pH 7.5), *S. boulardii* in 5% bicarbonate and HEPES buffer or *S. boulardii* in HEPES-buffered saline only. To further increase colonization, we also doubled the gavage dose of *S. boulardii* to 2×10^8^ CFU and repeated gavage every other day for 5 days. Higher levels of colonization in tissue were observed with each of these pH neutralization schemes (Fig. 4B). Compared to the lower dose of *S. boulardii* in PBS, the higher dose combined with pH neutralization increased colonization 30-fold (from 7×10^2^ to 2.3×10^4^ CFU/g tissue) in the cecum and 70-fold (from 2×10^2^ to 1.4×10^4^ CFU/g tissue) in the colon (Fig. 4A versus B). For simplicity of administration, subsequent experiments used HEPES-buffered saline alone.

### *In vivo* synthesis of VHH nanobodies by *S. boulardii*

As described above, we observed that *S. boulardii* fitness and VHH secretion level were impacted by the choice of promoter. To address whether these *in vitro* results extended to GI tract colonization *in vivo*, *S. boulardi*i expressing anti-mTNF VHH under the control of the *ADH2* or *ADH5* promoter, or *S. boulardii* with an empty plasmid vector or HEPES-buffered saline alone were gavaged for three successive days. Three days after the first gavage, VHH content was analyzed in the ileum, cecum, colon and feces by ELISA. *S. boulardii* that expressed anti-mTNF VHH from the *ADH2* promoter yielded higher VHH levels in all tissues analyzed, with a threefold increase in the colon compared to expression from the *ADH5* promoter (245±96.86 pg/ml for *ADH2* vs 79±30.48 pg/ml for *ADH5*) (Fig. 4C, right). This increased level of secretion was not due to differential colonization of the GI tract between the two strains (Fig. 4C, middle). We, therefore, prioritized the *ADH2*-driven anti-mTNF VHH-expression strains for subsequent testing in a mouse model of colitis.

A critical question for *in vivo* efficacy was whether the level of neutralizing VHH is comparable to the endogenous levels of mTNF found in the disease model. We, therefore, quantified relative anti-mTNF VHH and mTNF levels by ELISA, and converted these values to concentration estimates using standard curves with recombinant proteins. Because mTNF levels in the uninflamed colon were expected to be low, we assessed mTNF levels in the well-established DSS-induced mouse colitis model, which is widely used to evaluate anti-inflammatory therapeutic strategies in IBD (Lynch et al., 2023; Vandenbroucke et al., 2010; Wu et al., 2024). We found that anti-mTNF VHH levels in tissues from untreated animals, as driven by the *ADH2* promoter, exceeded the levels of mTNF (25-50 pg/100 μl TNF in feces or different tissues are equal to 5-10 pM of active TNF trimer, compared to 10-30 pM of anti-TNF VHH detected by ELISA in feces or different tissues) (see Fig. 4C versus D). These results suggested that strains producing anti-mTNF VHH have the potential to neutralize endogenous mTNF levels and remediate the GI inflammation observed in the DSS-induced model.

### Passage of *S. boulardii* through the GI tract does not affect VHH production or stability

An important question for any synthetic biotic is whether the intestinal environment selects against the production of the desired biologically active agent. To address this issue, we compared anti-mTNF VHH levels produced by an input *S. boulardii* strain to individual clones isolated from feces 2 days post gavage. We found no difference in VHH production between the input and output strains recovered after passage through the GI tract (Fig. S3A). This result suggested that counterselective pressure against VHH-producing *S. boulardii* in the GI tract is negligible. A further question was whether the secreted VHH is stable in the intestinal environment. To address this question, we assessed the stability of the anti-mTNF VHH when spiked into GI extracts. The anti-mTNF VHH was stable in feces, and cecum and colon extracts, but was degraded when incubated with an ileum extract, which is estimated to have 20-fold more proteolytic activity than feces (Macfarlane et al., 1988) (Fig. S3B). We concluded that the activity of *S. boulardii*-produced anti-mTNF VHH is unlikely to be impaired in the large intestine.

### DSS-induced colitis does not alter *S. boulardii* colonization or anti-mTNF VHH production

**Fig. 5.**
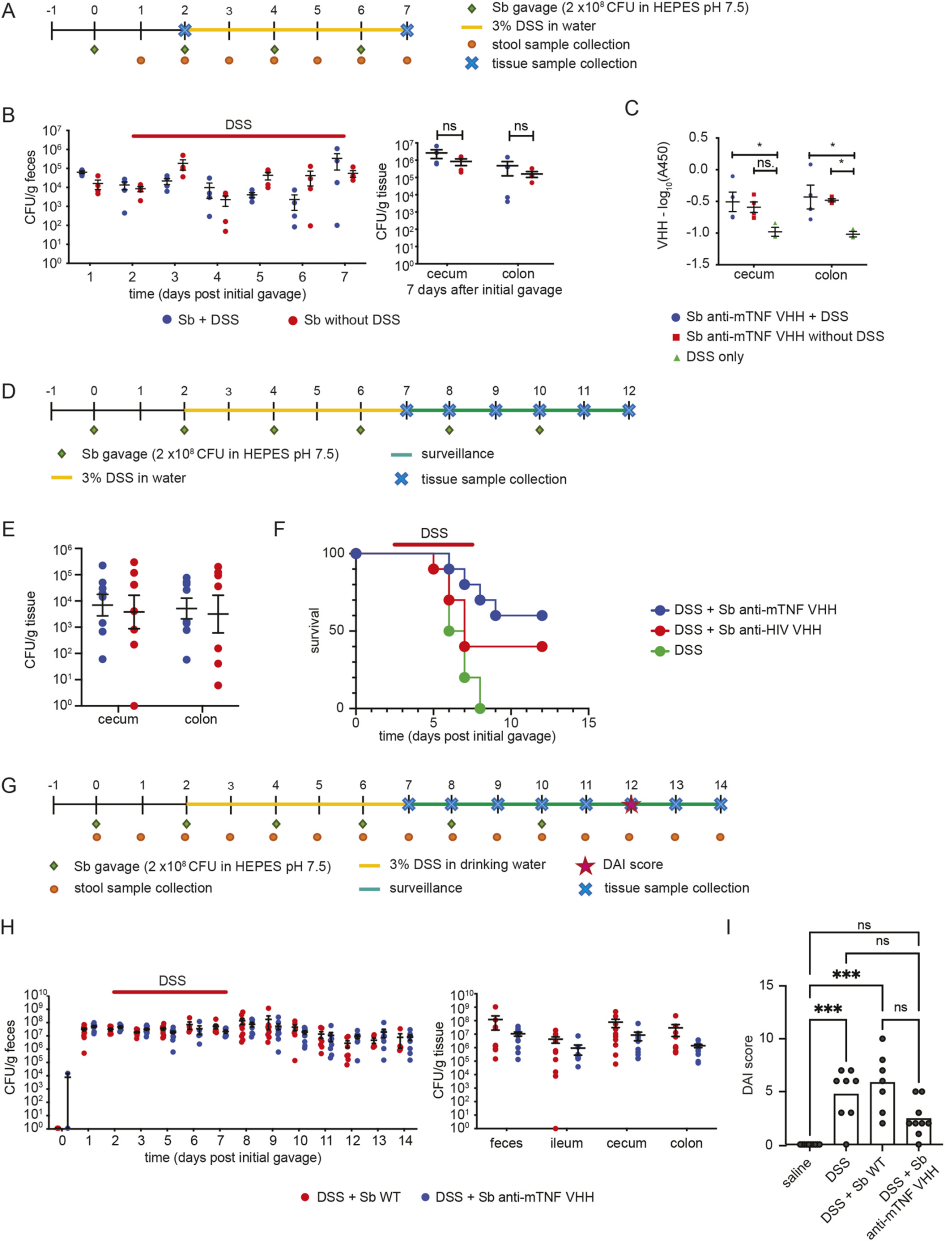
***In situ* production of anti-mTNF VHH by *S. boulardii* increases survival and improves symptoms in a DSS-induced colitis model.** (A) Schematic of experimental time course for *S. boulardii* (*Sb*) colonization and VHH production. (B) *S. boulardii* colonization with or without DSS-induced colitis in feces over 7-day time course (left) and in GI tissue (right) at 7-day experimental endpoint. Animals were pretreated with a single dose of 20 mg streptomycin 24 h prior to initial *S. boulardii* administration then gavaged once every 2 days with 2×10^8^ CFU wild-type *S. boulardii* or *S. boulardii* expressing anti-mTNF VHH from the *ADH2* promoter in HEPES-buffered-saline. CFUs were determined by serial dilution of feces at each time point or in indicated tissues at either humane (>20% weight loss) or experimental endpoint. Means±s.e.m. are shown. (C) Relative levels of anti-mTNF VHH in tissues at 7-day experimental endpoint was measured by ELISA. (D) Schematic of 12-day experimental time course for survival after DSS treatment. (E) *S. boulardii* colonization in GI tissue after DSS-induced colitis at 12-day experimental endpoint. Animals were pretreated with a single dose of 20 mg streptomycin 24 h prior to initial *S. boulardii* administration then gavaged once every 2 days with 2×10^8^ CFU *S. boulardii* expressing anti-mTNF VHH (m3F) or control anti-HIV-1 VHH (HIV-2) from the *ADH2* promoter in HEPES-buffered saline. CFUs were determined in tissues at either humane (>20% weight loss) or experimental endpoint. Each datapoint indicates an individual animal. Means±s.e.m. are shown. (F) Survival curves after DSS-induced colitis with administration of *S. boulardii* secreting either anti-mTNF VHH or control anti-HIV VHH versus HEPES-buffered saline alone. Survival was defined as weight loss of less than 20% initial body weight (mice that lost greater than 20% body weight were removed from the experiment and immediately euthanized). (G) Schematic of 14-day experimental time course for disease severity. Animals were pretreated with a single dose of 20 mg streptomycin 24 h prior to initial *S. boulardii* administration then gavaged once every 2 days with 2×10^8^ CFU wild-type *S. boulardii* or *S. boulardii* expressing anti-mTNF VHH (m3F) from the *ADH2* promoter in HEPES-buffered saline. CFU were determined by serial dilution of feces at each time point or in indicated tissues at either humane (>20% weight loss) or experimental endpoint. (H) *S. boulardii* colonization with or without DSS-induced colitis in feces (left) and GI tissue (right) at 14 day experimental endpoint. Means±s.e.m. are shown. (I) Disease activity index (DAI) scores after DSS-induced colitis with administration of *S. boulardii* secreting anti-mTNF VHH (m3F) or wild-type *S. boulardi* (WT) versus HEPES-buffered saline alone or HEPES-buffered saline without DSS treatment. Animals were pretreated with a single dose of 20 mg streptomycin 24 h prior to the first *S. boulardii* administration. DAI was scored at day 12 of the time course. DAI scores were: DSS+vehicle only, 5.43; DSS+wild-type *S. boulardii*, 5.86; DSS+*S. boulardii* anti-mTNF VHH (m3F) 2.44; saline, 0. Statistical analysis was calculated by one-way ANOVA, Kruskal–Wallis test, Dunn's multiple comparisons test (*P<0.05, ***P<0.001). Means±s.e.m. are indicated.

To assess efficacy *in vivo*, we evaluated the anti-mTNF VHH strain in the well-established DSS-induced mouse colitis model. DSS is a sulfated polysaccharide that is toxic to the colonic epithelium and, when administered to mice in drinking water, causes disruption of the intestinal–epithelial barrier as well as inflammation limited to the colon (Chassaing et al., 2014). We first determined whether DSS treatment altered *S. boulardii* colonization of the GI tract. We oral gavaged C57BL/6 mice once every two days with 2×10^8^ CFU of the anti-mTNF strain in HEPES-buffered saline for 7 days in the presence or absence of 3% DSS in drinking water (Fig. 5A). DSS did not cause any significant differences in CFU counts in either feces or colonic tissue (Fig. 5B). Although it has been previously shown that retention of *S. boulardii* in the mouse intestinal epithelium is increased by inflammation (Hudson et al., 2016), we did not observe any significant increase in *S. boulardii* CFUs within cecum and colon tissue upon DSS-induced inflammation. We next determined VHH levels in tissues at the experimental endpoint (i.e. day 7) of the DSS-treatment time course. Treatment with HEPES-buffered saline alone served as a control. The secreted anti-mTNF VHH was readily detected by ELISA in feces, as well as in cecum and colonic fractions (Fig. 5C). This result demonstrated that VHH secretion *in vivo* is not affected by DSS-induced colitis.

### *S. boulardii* expressing anti-mTNF VHH reduces disease severity in DSS-induced colitis

To assess whether *in situ* secretion of anti-mTNF can reduce mortality in the DSS-induced model, we carried out a 12-day experiment, in which animals were allowed to progress after DSS treatment (Fig. 5D). Animals were gavaged once every 2 days with 2×10^8^ CFU of *S. boulardii* in HEPES-buffered saline throughout the 12-day experiment and exposed to 3% DSS in drinking water between day 2 and 7, followed by 5 days without DSS exposure. Animals treated with a strain expressing the anti-HIV VHH or with HEPES-buffered saline only, were used as controls. No difference in *S. boulardii* retention was detected in GI tissues at the end of the experiment (day 12) (Fig. 5E). Administration of the anti-mTNF VHH strain increased survival of mice treated with DSS by 60% compared to administration of HEPES-buffered-saline alone (Gehan–Breslow–Wilcoxon test, *P*=0.0022) (Fig. 5F). The control VHH strain also conferred a survival benefit of 40% over HEPES-buffered saline alone, but this effect was lower than that for the anti-mTNF VHH strain (control VHH versus anti-mTNF VHH, *P*=0.0087, log-rank Mantel–Cox test). The partial effect of the control VHH strain we observed was consistent with the probiotic properties of *S. boulardii* reported for IBD (Dalmasso et al., 2006; Pothoulakis, 2009). To further characterize the effect of the anti-mTNF VHH strain, we performed a second recovery experiment over 14 days and monitored the disease activity index (DAI) with a wild-type strain as the control (Fig. 5G). As expected, no differences in colonization between strains were observed in feces or tissues (Fig. 5H). The anti-mTNF VHH strain improved the DAI score compared to that of the wild-type *S. boulardii* control strain and the HEPES-buffered saline control at the experimental end point in response to DSS treatment, but statistical significance was not reached. However, in the same experiment, the anti-mTNF VHH group treated with DSS was not statistically different from the untreated saline control group (*P*=0.1186, Dunn's multiple comparison test), whereas saline and wild-type *S. boulardii* DSS-treated controls were statistically worse than the untreated saline only control (Fig. 5I). Collectively, these results demonstrated that *in situ* production of the anti-mTNF VHH by *S. boulardii* have a pronounced beneficial effect on both survival and disease severity in the DSS-induced model.

### *S. boulardii* expressing anti-mTNF VHH does not alter the endogenous microbiome

**Fig. 6.**
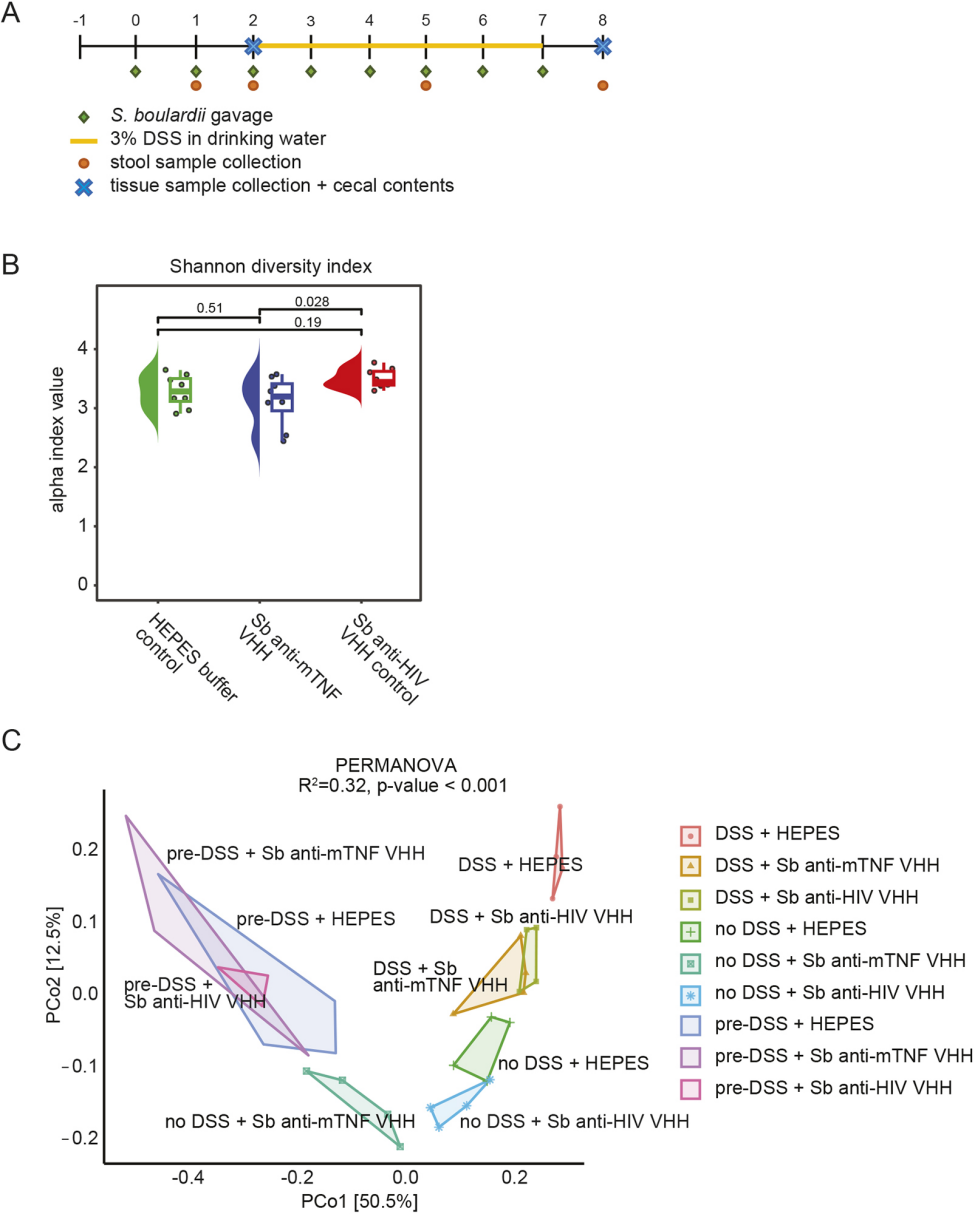
**Assessment of the endogenous GI microbiome in the presence or absence of *S. boulardii* expressing anti-mTNF VHH.** Cecal contents from animals administered the indicated *S. boulardii* strains (*Sb*) with or without DSS-induced colitis were sequenced in the V3-4 region of bacterial 16S rRNA. (A) Schematic of 8-day experimental time course. (B) Alpha diversity assessed by Shannon index. Statistical significance was assessed by ANOVA. (C) Beta diversity assessed by principal component analysis of amplicon sequence variants. Statistical significance was assessed by PERMANOVA.

Long-term *S. boulardii* administration in the mouse DSS-induced colitis model has been previously shown to increase bacterial diversity of the microbiome (Rodriguez-Nogales et al., 2018). We, thus, explored whether mice treated with *S. boulardii* that express anti-mTNF VHH exhibit alterations in the microbiome. Mice were gavaged daily with strains expressing anti-mTNF VHH or control anti-HIV VHH, or were treated with HEPES-buffered saline alone over 5 days DSS (Fig. 6A). Microbiome compositions were evaluated by 16S rRNA amplicon sequencing. As assessed by Shannon diversity index, which measures alpha diversity of the intestinal microbiome, we observed no significant difference between mice that were administered the buffer control and mice that had received strains expressing either VHH (Fig. 6B). Thus, the engineered probiotics did not influence the diversity of the intestinal microbiota. The similarity between microbiome composition (i.e. beta-diversity) of the different sample groups was then assessed using canonical correspondence analysis (CCA). Dispersion of beta diversity was computed using permutational multivariate analysis of variance (PERMANOVA). We observed that samples clustered together independently of the treatment when taken prior to colitis induction, after DSS treatment or at endpoint (Fig. 6C). Based on these results, we concluded that the beneficial effects of acute treatment with the anti-mTNF VHH strain are due to the anti-mTNF VHH itself and not a general effect of *S. boulardii* on the microbiome. We also concluded that short-term administration of the anti-mTNF VHH strain does not disrupt the overall composition of the microbiome.

## DISCUSSION

Wild-type *S. boulardii* has been extensively studied and used as a probiotic for the treatment of antibiotic-induced diarrhea, *C. difficile* infections, as well as in patients suffering from UC and CD (Kelesidis and Pothoulakis, 2012; Sivananthan and Petersen, 2018). The engineering of *S. boulardii* to deliver therapeutic biologics, such as nanobodies, directly to the GI tract has the potential to combine the general therapeutic effect of this commonly used yeast probiotic with highly targeted therapies (Chen et al., 2020; Heavey et al., 2022). Importantly, the use of *S. boulardii* as a living cell microfactory for *in situ* production of VHH nanobodies, may provide an accessible and cost-effective alternative to conventional recombinant biologic therapies that currently require systemic intravenous administration. Here, we demonstrate that anti-mTNF VHH nanobodies secreted in the GI tract can alleviate symptoms in a mouse model of colitis. Our platform was optimized for (i) VHH nanobody expression at low-nutrient conditions that mimic the GI environment, (ii) maximal levels of VHH nanobody secretion without causing proteostatic stress and, (iii) delivery regimes that maximize transient population of the GI tract. We specifically avoided the use of auxotrophic markers for strain engineering and, instead, used a fully prototrophic wild-type strain to maximize strain fitness within the GI environment – as enabled by a combination of CRISPR/Cas9-based genome engineering and the dominant counterselectable *amdS* marker (Stovicek et al., 2017). These features represent improvements over previous engineering methods in *S. boulardii* that relied on laterally transferrable plasmid vectors and dominant antibiotic resistance markers (Durmusoglu et al., 2021; Liu et al., 2016). Our results also underscore the importance of optimization for *in vitro* versus *in vivo* applications. Notably, while the *ADH2* and *ADH5* promoters yielded similar amounts of VHH in culture media, the *ADH2* promoter clearly outperformed *ADH5* for VHH production in the GI tract, despite the fact that no difference in tissue CFU levels was observed between the two strains.

Tandem bivalent or multivalent versions of VHH nanobodies can improve the affinity towards target epitopes. For example, high avidity nanobody multimers can neutralize influenza, SARS-CoV-2 and *C. difficile* (Chen et al., 2020; Laursen et al., 2018; Xiang et al., 2020). We tested several anti-human and anti-mouse TNF bivalent tandem VHH constructs but found that secretion of larger fusion proteins tended to reduce *S. boulardii* fitness, probably because of proteotoxic stress. While secretion of bivalent VHHs might be improved by engineering the *S. boulardii* protein-secretion system (de Ruijter et al., 2016; Huang et al., 2018)*,* modifications to *S. boulardii* need to be carefully assessed for impact on both secretion efficiency and fitness. Despite the close relationship between *S. boulardii* and *S. cerevisiae*, our secretion-signal optimization experiments demonstrated that improvements in *S. cerevisiae* do not necessarily translate to *S. boulardii*, as shown by the deleterious effects of deleting *HDA2*, *VPS5* and/or *TDA3* in *S. boulardii.* These unexpected negative effects may be because optimization of *S. cerevisiae* protein secretion has been largely explored in the context of improving yields of recombinant proteins under optimal growth conditions in fermenters. In the context of synthetic biotic development, *S. boulardii* secretion must be optimized for the relatively nutrient-poor conditions in the GI tract and for competition with the resident GI microbiome.

*S. boulardii* is generally recognized as a safe agent, and we observed no adverse effects upon oral administration of either wild-type or engineered *S. boulardii* to mice. In addition, we did not observe any significant changes in the microbiome associated with the acute administration of either wild-type or engineered *S. boulardii* strains. Further advantages of *S. boulardii* as a synthetic biotic platform include its relatively short residency time in the GI tract and the absence of horizontal gene transfer – a risk with bacterial synthetic biotics (Toth et al., 2021). Our results demonstrate the pre-clinical efficacy of engineered *S. boulardii* as a biotherapeutic production platform for anti-TNF VHH-based remediation of IBD, thereby positioning this approach for consideration in future human clinical trials.

The *S. boulardii* platform developed here can be readily adapted for the delivery of other therapeutic biologics. For example, *S. boulardii*-mediated secretion of neutralizing nanobodies directed at targets implicated in IBD, such as IL-12, IL-23, α4β7 integrin and/or α4β1 integrin may have advantages compared to systemic delivery of approved monoclonal antibodies against these targets (Zhou et al., 2015). This approach may be extended to *S. boulardii*-mediated delivery of nanobodies that neutralize bacterial antigens associated with IBD pathogenesis (Shaler et al., 2019). The levels of VHH achieved in the ileum also suggest that engineered *S. boulardii* strains may be used to deliver biologics that target diseases of the small intestine (Kerr et al., 2011). The *S. boulardii* platform may also be readily deployed for veterinary applications, for instance, in the prevention and treatment of various diseases and infections in livestock. *S. boulardii* probiotics are already approved for use in livestock to increase production, reduce cost and mitigate dependence on antibiotics (Daudelin et al., 2011; Foo et al., 2017; Hancox et al., 2015; Massacci et al., 2019; O'Callaghan et al., 2016). Further development of the *S. boulardii* platform holds promise for low-cost production and facile oral delivery of highly specific biologic agents in manifold contexts.

## MATERIALS AND METHODS

### Plasmid construction

All constructs consisted of a promoter, secretion signal, antibody, secretion tag, *CYC1* terminator, selection marker, 2-µm origin of replication, ampicillin resistance gene and *E. coli* origin (see Fig. S4A for plasmid map). Promoters were amplified from *S. cerevisiae* or *S. boulardii*, secretion signals (Table S1) and nanobodies (Table S2) were obtained as gBlocks (Integrated DNA Technologies). Antibody sequences were as reported (Chen et al., 2019; Wong, 2013). Plasmids were constructed using Gibson Assembly cloning (Gibson et al., 2009). To generate gene knockouts, plasmid pGZ110 that expresses SpCas9 and a targeting sgRNA (gift from Bruce Futcher, SUNY Stony Brook, NY, USA) was modified to replace the *URA3* auxotrophic selection marker with the *amdS* recyclable selectable marker (Fig. S4), which confers resistance to acetamide and can be counterselected using fluoroacetamide (Solis-Escalante et al., 2013). Guides to target *URA3*, *HDA2*, *TDA3* and *VPS5* were designed by using the Synthego Knockout Guide Design tool (https://design.synthego.com/#/). The vector also contained a DNA donor template for homology-directed repair that introduced several stop codons and a frameshift to prevent read-through into each coding region, as well as a protospacer adjacent motif (PAM) site deletion to prevent continuous Cas9 nucleolytic activity (see Table S3 for sequences). After homozygous deletion was confirmed by Sanger sequencing (Fig. S2A), the plasmid was removed by counterselection on fluoroacetamide-containing medium. All plasmids used in this work are listed in Table S4 and are available upon request.

### Yeast strain construction

Yeast cells were transformed using the lithium acetate method (Gietz et al., 1992). *S. boulardii* (MYA-796, obtained from ATCC) and *S. cerevisiae* (Sigma1278b) were inoculated overnight at 30°C while shaking at 220 rpm, diluted to an OD_595_ of 0.5, and incubated at 30°C while shaking at 220 rpm until an OD_595_ of 1.5 was reached. Cells were then washed with distilled sterile water, resuspended in transformation mix (240 µl 50% PEG 3350, 36 µl 1 M lithium acetate, 50 µl single-stranded carrier DNA (2 mg/ml in TE), 36 µl 1 M DTT, and 16 µl water+plasmid DNA (1 µg in *S. boulardii* and 100 ng in *S. cerevisiae*). Cells were incubated at 42°C for 40 min, pelleted and washed with 1 ml sterile water, resuspended in 200 µl water and immediately plated onto appropriate selective medium. For G418 selection cells were recovered overnight in XY-rich medium [yeast extract peptone dextrose (YEPD)+0.01% w/v adenine and 0.02% w/v tryptophan] at 30°C and 220 rpm. The transformation efficiency of *S. boulardii* was typically 30-50 times below that for *S. cerevisiae* (Liu et al., 2016). For *URA3* selection, cells were directly plated on SC-Ura medium. All strains used in this work are listed in Table S5 and are available upon request.

### Antibody and nanobody detection

Cells expressing antibody fragments or VHH nanobodies under the control of the *GAL1* promoter were precultured overnight in XY+2% raffinose at 30°C, diluted 200-fold into XY+2% galactose for 2-4 days. Cells expressing antibodies or nanobodies under the control of constitutive promoters were grown for 3 days in XY+2% glycerol+ 2% ethanol or XY+glucose at the indicated concentrations and at the indicated temperatures. Cultures were centrifuged, supernatant collected and pellets washed with cold water. Proteins in the culture medium were precipitated in 10% TCA dissolved in acetone at a 1:10 ratio. Cell pellets were dissolved with 2 M LiOAc and 0.4 M NaOH for 5 min in each solution (Zhang et al., 2011). For immunoblot analysis, antibodies and nanobodies were separated by SDS-PAGE containing 15% acrylamide (VHH) or 12% acrylamide (Fab′) (Bio-Rad) and then transferred to a nitrocellulose membrane using a Wet/Tank Blotting System (Bio-Rad) or directly stained with Coomassie Blue. Membranes were probed with mouse monoclonal anti-FLAG-HRP (Sigma A8592) at 1:5000 dilution or anti-VHH-HRP (GenScript A01861) at 1:3000 dilution, and signals were visualized by chemiluminescence (Western Lightning Plus-ECL) using a ChemiDoc imaging system (BioRad).

### Growth rate analysis

A preculture was grown overnight until saturation in XY+2% ethanol+ 2%glycerol or 2% glucose and then diluted 20-fold in 96-well clear flat-bottomed microplates (Corning Costar) in the same medium. The plates were sealed and incubated for 72 h on a Sunrise shaker-reader (Tecan) at 37°C with shaking at 564 rpm. Absorbance at 595 nm was measured every 15 min for 24 h. Growth rates were calculated as the linear portion of the curve by using PRISM software (GraphPad).

### VHH nanobody purification

Cells were grown in XY+2% ethanol+2% glycerol for 3 days in 500 ml culture at 30°C. The culture was centrifuged at 3000 rpm and the supernatant adjusted to pH 8.0 with 10 N NaOH. The supernatant was applied on a His-Trap HP column (GE Healthcare), washed with buffer (50 mM Tris pH 8.0, 120 mM NaCl, 10% glycerol, 20 mM imidazole), and eluted with wash buffer containing 500 mM imidazole. Eluted fractions were concentrated by ultrafiltration using a 3 kDa cut-off spin filter (Vivaspin). Quantification of VHH abundance was performed using a Pierce 660 nm colorimetric assay (Thermo Fisher Scientific).

### Direct ELISA

For proteins in yeast culture medium, 100 ng of either mTNF or mouse monoclonal anti-VHH in phosphate-buffered saline (PBS) were incubated for 2 h at 4°C on protein-binding Nunc MaxiSorp^®^ flat-bottom 96-well plates and then blocked with 100 µl of 0.1 M NaHCO_3_ pH 8.6+2% BSA for 1 h at room temperature (RT). The protein of interest was diluted in PBS and incubated for 1 h at RT, followed by incubation with anti-FLAG-HRP or anti-HIS-HRP (1:5000 in PBS) for 1 h at RT and subsequent development with 50 µl of 3,3′,5,5′-tetramethylbenzidine (TMB, R&D Systems #555214) substrate. The reaction was stopped with 100 µl of 1 M sulfuric acid and absorbance was read at 450 nm on a microplate reader (Tecan) or on an Envision microplate reader (Perkin Elmer).

For mouse tissues, Nunc MaxiSorp^®^ flat-bottom 96-well plates were coated overnight at 4°C with 100 ng of the protein of interest in PBS, and then blocked with 100 µl of ELISA/ELISPOT diluent (eBioscience). To generate tissue samples, female C57BL/6N (Charles River) 6- to 8-week-old mice were administered 20 mg streptomycin via oral gavage followed by doses of saturated *S. boulardii* yeast cultures via oral gavage according to the indicated regime for each experiment. Fecal pellets and tissues, including luminal contents, were homogenized in 1 ml PBS using a Mixer Mill MM 400 (RM400) homogenizer (Retsch, Haan, Germany). Samples were centrifuged and the amount of mF3 VHH nanobody in supernatants was quantified using a standard curve generated with purified m3F-FLAG VHH. For ELISA, an anti-FLAG capture antibody (Genescript #A00170) was used at 1:4000 dilution, followed by an anti-VHH-HRP detection antibody (Genescript #A01861) at 1:4000. ELISAs were developed using TMB (R&D Systems #555214) and read at 450 and 570 nm using an Envision plate reader (Perkin Elmer).

### Nanobody toxicity assays

T98G cells (ATCC) were grown in Dulbecco's modified Eagle's medium supplemented with 100 units/ml penicillin, 100 μg/ml streptomycin and 10% heat-inactivated fetal bovine serum (FBS). Cells were maintained at 37°C in 5% CO_2_ and under 100% humidity. For cytotoxicity assays, cells were seeded in 384-well plates for 24 h, followed by VHH addition using an Echo 555 acoustic dispenser (Labcyte). Cell viability was evaluated after 48 h by Cell Titer Glo in a Tecan reader (luminescence, gain 135, integration time 40 s). To evaluate the neutralization potential of different VHH preparations, a combination of actinomycin D (40 nM) and hTNF (500 ng/ml) was empirically chosen to provide an optimal dynamic range for the assay. Cells were treated with 500 ng/ml hTNF and 40 nM actinomycin D in the presence of increasing concentrations of the VHH purified from yeast culture medium. Neutralization activity was calculated by setting cell viability in actinomycin D as 1 and cell viability in actinomycin D+hTNF as 0.

### Colonization of mice with *S. boulardii*

All mouse experiments were performed in the Central Animal Facility at McMaster University under animal use protocol 20-12-41 as approved by the Animal Research Ethics Board, and under compliance with Canadian ethical regulations. Female C57BL/6N mice (Charles River, 027) aged 6-10 weeks were used in experiments. One day prior to *S. boulardii* administration, groups of two to five mice were pretreated with a single dose of 20 mg of streptomycin as indicated. When indicated, pretreatment with 5% NaHCO_3_ was carried out 30 min prior to yeast gavage (*S. boulardii* bearing *ADH2*-m3F VHH or *ADH2* control HIV-2 VHH). Mice were administered 2×10^8^ CFU (except for Fig. 4A in which 1×10^8^ CFU were administered) of the appropriate yeast suspension in either HEPES-buffered saline (0.9% NaCl, 100 mM HEPES pH 7.5) or 5% NaHCO_3_ with HEPES buffer via oral gavage at the indicated frequency for each experiment. In all cases, the wild-type *S. boulardii* control strain harbored an empty plasmid vector to enable detection and quantification of yeast CFUs. Fecal pellets were collected at the times indicated and homogenized in 1 ml PBS for 5 min at 30 rps (Retsch MM400) for determination of yeast CFU by serial dilution. At the experimental endpoint, ileum, cecum and colon tissue, including luminal contents, were collected and homogenized in 1 ml PBS for 10 min at 30 rps. All homogenates were serially diluted and plated on YPD containing 200 μg/ml G418 and 100 μg/ml ampicillin for CFU determination.

### DSS-induced colitis model

Two days following the initial gavage of *S. boulardii* strains or control buffer, mice were administered 2-3% sterile DSS (Thermo Fisher Scientific, CAS: 9011-18-1, #J1448922) in double distilled water *ad libitum*. After 5 days, mice were shifted to regular drinking water. Mice were euthanized after having reached either 20% weight loss or experimental endpoint (up to 2 weeks post initial gavage). Fecal pellets and tissue were collected and processed as above. Disease activity index (DAI), concordant with clinical symptoms observed in human IBD, was scored as described previously (Yao et al., 2010). Briefly, DAI scores were determined by evaluating for body weight loss, stool consistency and occult/gross bleeding, as stratified in Table S6. Statistical analyses were performed using GraphPad Prism v.98. Significance was assessed using the tests indicated for each experiment.

### 16S rDNA microbiome sequencing

Genomic DNA was extracted as described with modifications (Stearns et al., 2015). Samples were transferred to screw-cap tubes containing 2.8-mm ceramic beads, 0.1-mm glass beads, 5 M guanidinium thiocyanate, 0.1 M EDTA, 0.5% sarcosyl, and sodium phosphate buffer as described (Stearns et al., 2015). Samples were homogenized with a bead beater, centrifuged and the supernatant was processed using a MagMAXExpress 96-Deep Well Magnetic Particle Processor (Applied Biosystems) with a Multi-Sample kit (Life Technologies, #4413022). Purified DNA was used to amplify variable regions 3 and 4 of the 16S rRNA gene by two-stage nested PCR. First, samples were amplified in the 8f (5′-AGAGTTTGATCCTGGCTCAG-3′) to 926r (5′-CCGTCAATTCCTTTRAGTTT-3′) region of the 16S gene. The reaction was carried out at 94°C for 5 min, 15 cycles at 94°C for 30 s, 56°C for 30 s and 72°C for 60 s, with a final extension at 72°C for 10 min. PCR products were used as template in the second stage of PCR, in which the 341F (5′-CCTACGGGNGGCWGCAG-3′) to 806R (5′-GGACTACNVGGGTWTCTAAT-3′) region was amplified as previously described (Bartram et al., 2011). The reaction was carried out at 94°C for 5 min, with five cycles at 94°C for 30 s, 47°C for 30 s and at 72°C for 30 s, followed by 25 cycles at 94°C for 30 s, 50°C for 30 s and 72°C for 30 s, with a final extension at 72°C for 10 min. Resulting PCR products were visualized on a 1.5% agarose gel. Positive amplicons were pooled to normalize based on band intensity and sequenced on an Illumina MiSeq platform.

Bioinformatic analysis was performed as previously described (Barot et al., 2024; Chambers et al., 2022; Weiss et al., 2021). Briefly, raw 16S amplicon sequences and metadata were demultiplexed using *split_libraries_fastq.*py script implemented in *QIIME2* (Bolyen et al., 2019)*.* Demultiplexed fastq files were split into sample-specific fastq files using *split_sequence_file_on_sample_ids*.py script from *QIIME2*. Individual fastq files without non-biological nucleotides were processed using Divisive Amplicon Denoising Algorithm (DADA) pipeline (Callahan et al., 2016). The output of the DADA2 pipeline (feature table of amplicon sequence variants) was processed for alpha and beta diversity analysis using *phyloseq* (McMurdie and Holmes, 2013), and *microbiomeSeq* (http://www.github.com/umerijaz/microbiomeSeq) packages in R. We analyzed variance (ANOVA) among sample categories while measuring the of α-diversity measures using *plot_anova_diversity* function in *microbiomeSeq* package. Permutational multivariate analysis of variance (PERMANOVA) with 999 permutations was performed on all principal coordinates obtained using canonical correspondence analysis (CCA) with the *ordination* function of the *microbiomeSeq* package.

### Statistical analyses

All statistical tests were performed with GraphPad Prism software (www.graphpad.com). Expression analysis of the different promoters and constructs in *S. boulardii* was performed using ANOVA Turkey's multiple comparison. DAI score statistical analysis was performed using ANOVA, Kruskal–Wallis test, and Dunn's multiple comparison. Statistical significance of the survival data was analyzed using Gehan–Breslow–Wilcoxon. All *P*-values are listed in figure legends.

## Supplementary Material

10.1242/dmm.052620_sup1 Supplementary information
