# Supplementary material for: A yeast synthetic biotic platform for delivery of therapeutic nanobodies to ameliorate gastrointestinal inflammation
Source: Dis Model Mech. 2026 Mar 9;19(2):dmm052620. doi: 10.1242/dmm.052620 (PMC13035066; doi:10.1242/dmm.052620)
Supplement: Supplementary information [file dmm-19-052620-s1.pdf]

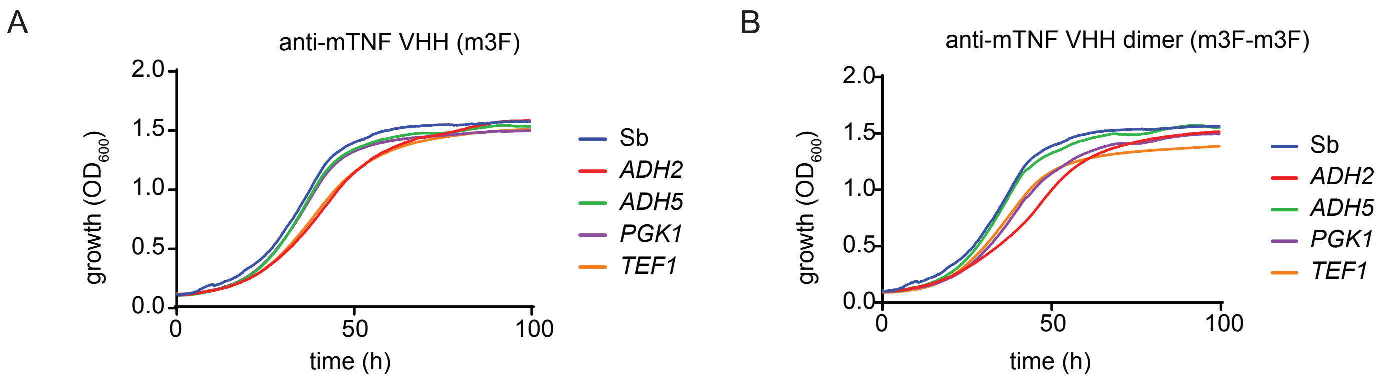


**Fig. S1. Effect of anti-mTNF VHH on *S. boulardii* proliferation *in vitro*. A)** Growth rate of strains producing anti-mTNF VHH (m3F) produced under the control of *ADH2*, *ADH5*, *PGK1* and *TEF1* promoters were determined by $OD_{595}$ in a shaker-reader at 37°C. **B)** Growth rate of strains producing anti-mTNF VHH tandem dimer (m3F-m3F) produced under the control of *ADH2*, *ADH5*, *PGK1* and *TEF1* promoters were determined by $OD_{595}$ in a shaker-reader at 37°C. Wild type S. *boulardii* (Sb) was used as a control.

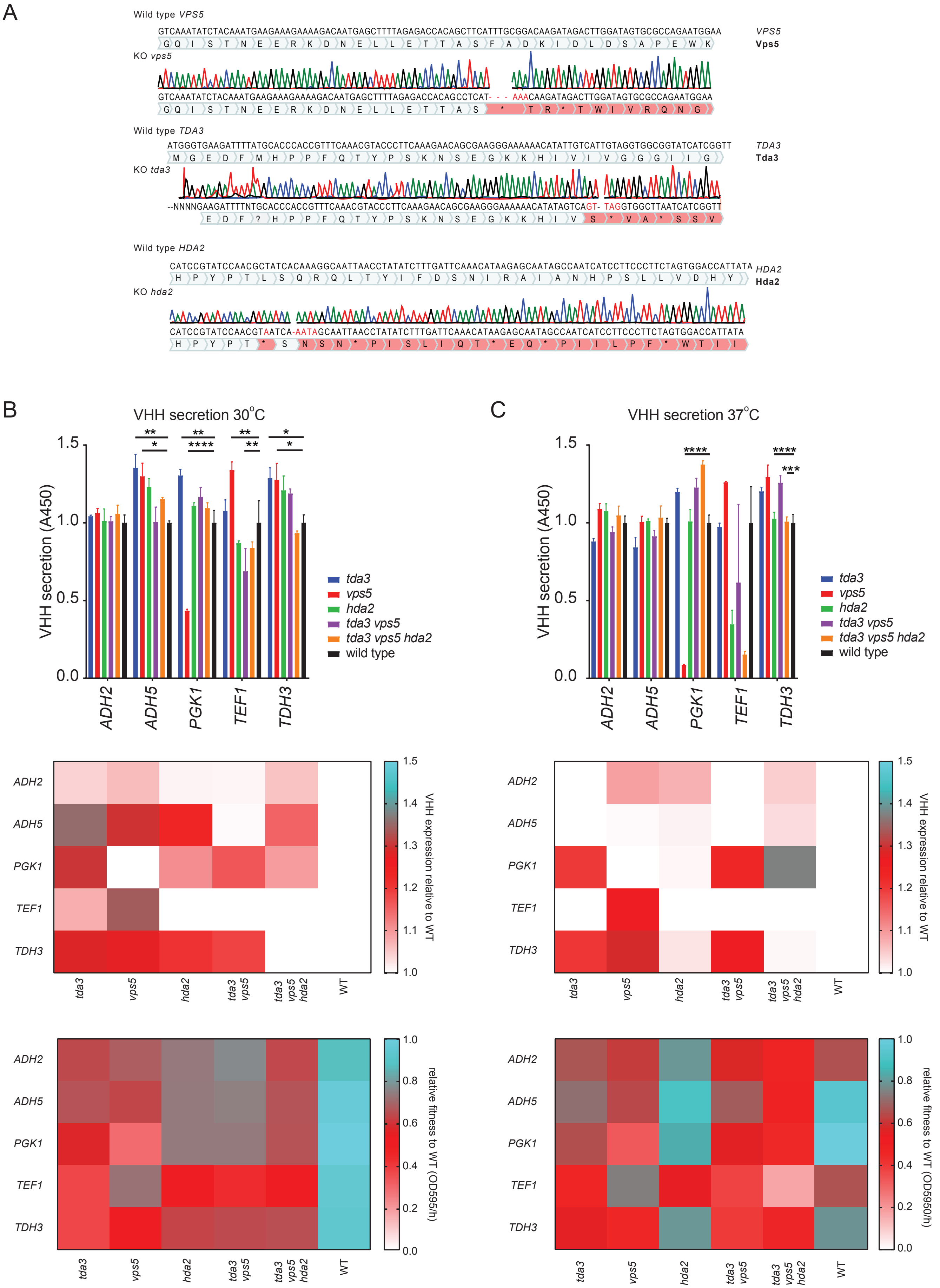
A
Wild type VPS5
VPS5
Vps5
KO vps5
Wild type TDA3
TDA3
Tda3
KO tda3
Wild type HDA2
HDA2
Hda2
KO hda2
B
VHH secretion 30°C
C
VHH secretion 37°C
VHH secretion (A450)
1.5
1.0
0.5
0.0
ADH2
ADH5
PGK1
TEF1
TDH3
tda3
vps5
hda2
tda3 vps5
tda3 vps5 hda2
wild type
VHH expression relative to WT
relative fitness to WT (OD595/h)
relative fitness to WT (OD5950/h)
WT

**Fig. S2. Effect of secretory pathway mutations on VHH production by *S. boulardii*.** The secretory pathway genes *TDA3, VPA5* and *HDA2* were disrupted by CRISPR-mediated introduction of frameshift mutations. **A)** Sequence validation of mutant alleles. Sequences were rendered and visualized in Benchling. **B)** Single, double, and triple mutants were transformed with plasmids expressing anti-mTNF VHH (m3F) under the control of the *ADH2*, *ADH5*, *PGK1*, *TEF1* and *TDH3* promoters. Cells were grown in XY rich medium with 2% glycerol+2% ethanol at 30°C (*left*) in a shaker-reader for 3 days. VHH secretion was quantified by ELISA using immobilized anti-VHH antibody (top) and normalized to wild type *S. boulardii* expressing anti-mTNF m3F VHH from the *PGK1* promoter (middle). Mean ± SEM are indicated. Statistical analysis by Tukey's ANOVA (* $p<0.05$, ** $p<0.01$, *** $p<0.001$, **** $p<0.0001$). Cell fitness was measured as the slope of the exponential growth phase for three independent colonies and normalized to wild type *S. boulardii* expressing anti-mTNF (m3F) VHH from the *ADH2* promoter (bottom). **C)** As for panel B but with cell culture at 37°C.

Disease Models & Mechanisms • Supplementary information

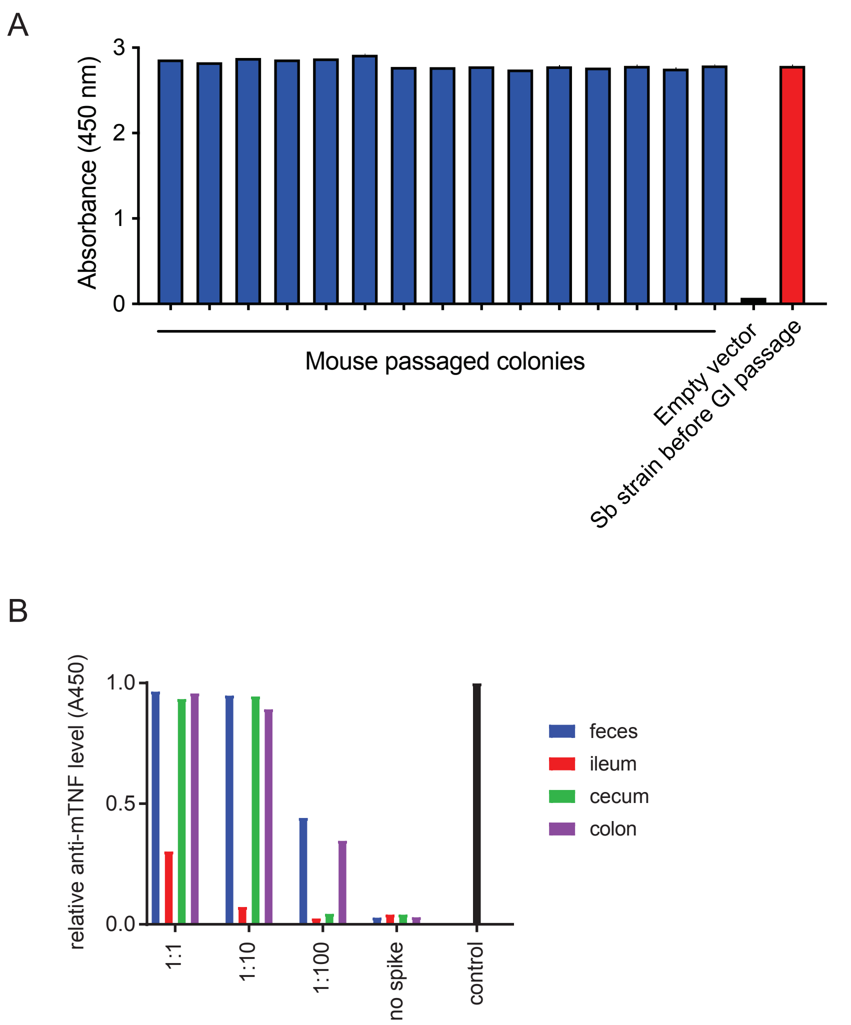


**Fig. S3. Production of anti-mTNF VHH by *S. boulardii* after passage through the mouse GI tract. A)** Individual *S. boulardii* colonies obtained from feces were assessed for anti-mTNF VHH (m3F) expression by ELISA. **B)** Stability of anti-mTNF m3F VHH activity in indicated tissue and feces extracts. Dilutions of anti-mTNF VHH produced by *S. boulardii* in culture medium were incubated with tissue extracts and assessed for binding activity by ELISA.

Disease Models & Mechanisms • Supplementary information

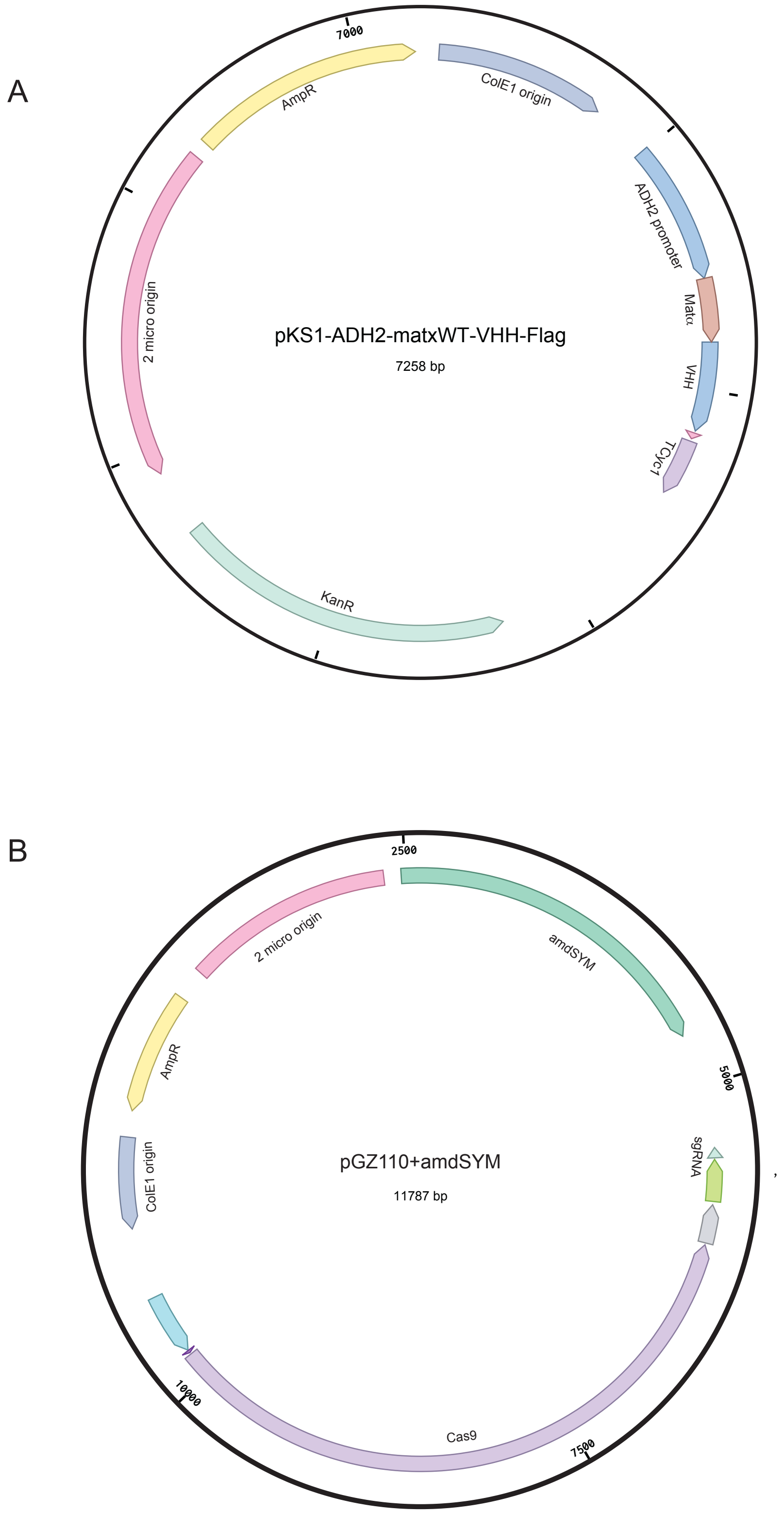


**Fig. S4. Plasmid map for constructs used in this study. A)** pKS-p*ADH2*-*MAT*aWT-VHH-FLAG-*CYC1*term-KanMX for VHH expression and secretion. Variations of this construct replaced promoters, secretion signals and VHH coding regions. **B)** pGZ110-Cas9-amdSYM for *S. boulardii* genome engineering. Variations of this construct replaced sgRNA and repair template regions.

Disease Models & Mechanisms • Supplementary information

## Table S1. List of secretion signals

| | Secretory Peptide Sequence | Origin |
|---|---|---|
| Amylase | MVAWWSLFLYGLQVAAPALA | (Paifer *et al.*, 1994) |
| Glucanase | MQRPFLLAYLVLSLLFNSAL | (Achstetter *et al.*, 1992) |
| Inulase | MKFAYSLLLPLAGVSA | (Massahi and Çalik, 2015) |
| Invertase | MLLQAFLFLLAGFAAKISA | (Chitoshi OKA *et al.*, 1999) |
| Lysozyme | MRSLLILVLCFLPLAALG | (Chitoshi OKA *et al.*, 1999) |
| Mat α WT | MRFPSIFTAVLFAASSALAAPVNTTTEDETAQIPAEAVIGYLDLEGDFDVAVLPFSNS TNNGLLFINTTIASIAAKEEGVQLDKR | |
| Mat α App8 (Ala22) | MRFPSIFTAVLFAASSALAAP**A**NTTTEDETAQIPAEAVI**D**Y**S**DLEGDFD**A**A**A**LP**L**SNS TNNGL**SST**NTTIASIAAKEEGVQLDKR | (Rakestraw *et al.*, 2009) |
| Mat α App8 A22V | MRFPSIFTAVLFAASSALAAPVNTTTEDETAQIPAEAVI**D**Y**S**DLEGDFD**A**A**A**LP**L**SNS TNNGL**SST**NTTIASIAAKEEGVQLDKR | (Rakestraw *et al.*, 2009) |
| BBa_K416 003 (BBaK) | MKVLIVLLAIFAALPLALAQPVISTTVGSAAEGSLDKR | (Clements *et al.*, 1901) |

* mutated residues in red with respect to the wild type Mata signal

## Table S2. List of VHH nanobody sequences

| Name | Code | Target | Sequence | Origin |
|---|---|---|---|---|
| HIV-1 | L8Cj3 | HIV-1 | VQLVESGGGLVQAGGFLRLSCELRGSIFNQYA MAWFRQAPGKEREFVAGMGAVPHYGEFVKGR FTISRDNAKSTVYLQMSSLKPEDTAIYFCARSKS TYISYNSNGYDYWGRGTQVTVSSGGSDYKDD DDK | (McCoy *et al.*, 2012) |
| HIV-2 | J3r | HIV-1 | VQLQESGGGLVQAGGSLRLSCELRGSIFNQYA MAWFRQAPGKEREFVAGMGAVPHYGEFVKGR | (McCoy *et al.*, 2012) |

Disease Models & Mechanisms • Supplementary information

| | | | | |
|---|---|---|---|---|
| | | | FTISRDNAKSTVYLQMNSLKPEDTAIYFCARSKSTYISYNSNGYDYWGQGTQVTVSSGGSDYKDDDDK | |
| HIV-3 | L911F1F | HIV-1 | VQLVESGGALVQAGRSLRLSCAASGNAFTIDAAAWYRQAPGKQREPVATILSGGTTNYADSVKGRFTISRDNVKNTVYLQMNSLKPEDTAVYYCYVPMVYYSGRYNDVWGQGTQVTVSSGGSDYKDDDDK | (McCoy *et al.*, 2012) |
| CD-1 | A4.2 | TcdA | EGVQLDKREAQVKLEESGGGLVQAGGSLRLSCAASGRTFNTLSMGWFRQAPGKEREFVAAVSRSGGSTYYADSVKGRFTISRDNAKNTVYLQMNSLKPEDTAVYYCAAAATKSNTTAYRLSFDYWGQGTQVTVSSGGSDYKDDDD | (Hussack *et al.*, 2011) |
| CD-2 | A20.1 | TcdA | EGVQLDKREAQVQLVESGGGLAQAGGSLRLSCAASGRTFSMDPMAWFRQPPGKEREFVAAGSSTGRTTYYADSVKGRFTISRDNAKNTVYLQMNSLKPEDTAVYYCAAAPYGANWYRDEYDYWGQGTQVTVSSGGSDYKDDDD | (Hussack *et al.*, 2011) |
| CD-3 | A26.8 | TcdA | EGVQLDKREAQVKLEESGGGLVQAGGSLRLSCAASERTFSRYPVAWFRQAPGAEREFVAVISSTGTSTYYADSVKGRFTISRDNAKVTVYLQMNNLKREDTAVYFCAVNSQRTRLQDPNEYDYWGQGTQVTVSSGGSDYKDDDD | (Hussack *et al.*, 2011) |
| CD-4 | A5.1 | TcdA | EGVQLDKREAQVKLEESGGGLVQAGGSLRLSCAASGRTFSMYRMGWFRQAPGKEREFVGVITRNGSSTYYADSVKGRFTISRDNAKNTVYLQMNSLKPEDTALYYCAATSGSSYLDAAHVYDYWGQGTQVTVSSGGSDYKDDDD | (Hussack *et al.*, 2011) |
| CD-5 | 4NC2 | TcdB | EGVQLDKREAQVQLVESGGGLVQAGGSLRLSCAASGLTFSRYVMGWFRQAPGKEREFVAAITWGGTPNYADSVKGRFTISRDNSKNTQYLQMNSLKPEDTAVYYCAAGLGWDSRYSQSYNYWGQGTQVTVSSGGSDYKDDDD | (Murase *et al.*, 2014) |
| CJ-1 | V1Flag | *C. jejuni* | EGVQLDKREAQVKLEESGGGLVQAGGSRRLSCATSGLTFRNFHMAWFRQVAGKEREVVAAISWSRDRQYYPDPVKGRFTITRDNAKNTVYLQMNSLKPEDTAVYYCAARTASASGDWYKGSYQYWGQGTQVTVSSGGSDYKDDDD | (Vanmarsenille *et al.*, 2017) |
| CJ-2 | V6Flag | *C. jejuni* | EGVQLDKREAQVKLEESGGGLVQAGGSLRVSCTASVSTFSINALGWYRQAPGKARELVAAIGSDGTVYYTDSVKGRFTISRDNAKNTVSLQMSSLKPE | (Vanmarsenille *et al.*, 2017) |

Disease Models & Mechanisms • Supplementary information

| | | | | |
|---|---|---|---|---|
| | | | DTAVYYCNAAGKRIGSDGSIWFAVASFGSWGQGTQVTVSSGGSDYKDDDD | |
| ETEC-1 | K922 | F4 fimbrae | EGVQLDKREAQVQLQESGGGLVQPGGSLRLSCLVSGGTFSWYAMGWFRQAPGKEREFVATVSRGGGSSYYADSVKGRFTISRDNAKNTVYLQMNSLKPEDTAVYYCAAGRGAPSDTGRPDEYDYWGQGTQVTVSSGGSDYKDDDD | (Harmsen *et al.*, 2006) |
| ETEC-2 | 4PC2 | Stxe2 | EGVQLDKREAQVQLQESGGGLVQAGGSLRLSCAVSGSIFRLSTMGWYRQAPGKQREFVASITSYGDTNYRDSVKGRFTISRDNAKNTVYLQMNSLKPEDTAVYYCNANIEAGTYYGPGRDYWGQGTQVTVSSGGSDYKDDDD | (Tremblay *et al.*, 2013) |
| LM-1 | VH303 | *L. monocytogenes* | EGVQLDKREAEVQLVESGGGLVQPGRSLRLSCAASGHTYSTYCMGWVRRAPGKGEELVARINVGGSSTWYADSVKGRFTISADTSKNTAYLQMNSLRAEDTAVYYCTLHRFCNTWSLGTLNYSGQGTLVTVSSGGSDYKDDDD | |
| LM-2 | VH-1bag | *L. monocytogenes* | EVQLVESGGGLVQPGRSLRLSCAASGFNIKDTYIGWVRRAPGKGEELVARIYPTNGYTRYADSVKGRFTISADTSKNTAYLQMNSLRAEDTAVYYCARWGGDGFYAMDYWGQGTLVTVSSGGSDYKDDDD | |
| hTNF-1 | 5M2I | human TNF | EGVQLDKREAQVQLVESGGGLVQAGGSLSLSCSASGRSLSNYYMGWFRQAPGKERELLGNISWRGYNIYYKDSVKGRFTISRDDAKNTIYLQMNRLKPEDTAVYYCAASILPLSDDPGWNTYWGQGTQVTVSSGGSDYKDDDD | (Beirnaert *et al.*, 2017) |
| hTNF-2 | 5M2J | human TNF | EGVQLDKREAQVQLVESGGGLVQPGGSLRLSCAASGFTFSNYWMYWVRQAPGKGLEWVSEINTNGLITKYPDSVKGRFTISRDNAKNTLYLQMNSLKPEDTALYYCARSPSGFNRGQGTQVTVSSGGSDYKDDDD | (Beirnaert *et al.*, 2017) |
| hTNF-3 | 5M2M | human TNF | EGVQLDKREAQVQLQESGGGLVQPGGSLRLSCAASGRTFSDHSGYTYTIGWFRQAPGKEREFVARIYWSSGNTYYADSVKGRFAISRDIAKNTVDLTMNNLEPEDTAVYYCAARDGIPTSRSVESYNYWGQGTQVTVSSGGSDYKDDDD | (Beirnaert *et al.*, 2017) |
| hTNF-4 | 5M2M-5M2I | human TNF | EGVQLDKREAQVQLQESGGGLVQPGGSLRLSCAASGRTFSDHSGYTYTIGWFRQAPGKEREFVARIYWSSGNTYYADSVKGRFAISRDIAKNTVDLTMNNLEPEDTAVYYCAARDGIPTSRSVESYNYW | (Beirnaert *et al.*, 2017) |

Disease Models & Mechanisms • Supplementary information

| | | | | |
|---|---|---|---|---|
| | | | GQGTQVTVGGGSGGGSGGGSQVQLVESGGGLVQAGGSLSLSCSASGRSLSNYYMGWFRQAPGKERELLGNISWRGYNIYYKDSVKGRFTISRDDAKNTIYLQMNRLKPEDTAVYYCAASILPLSDDPGWNTYWGQGTQVTVSSGGSDYKDDDD | |
| mTNF | M3F | mouse TNF | QVQLQDSGGGLVQAGGSLRLSCAASGGTFSSIIMAWFRQAPGKEREFVGAVSWSGGTTVYADSVLGRFEISRDSARKSVYLQMNSLKPEDTAVYYCAARPYQKYNWASASYNVWGQGTQVTVSSEPKTPKPQPVTVSSGGSDYKDDDDK | (Coppieters *et al.*, 2006) |

## Table S3. List of homology repair and sgRNA sequences

| Target | Homology | sgRNA |
|---|---|---|
| URA3 | AGTCCTGTTGCTGCCAAGCTATTTAATATGTTGCACGAAAAGCAAACAAACTTGTGTGCTTAATTGGATGTCCGTACCACGTAATAATACTGGAGTTAGTTGAAGCATTAGGTCCCAAAATTTGTTTACTAAAAACACATGTGGAT | ATTGGATGTTCGTACCACCA |
| HDA2 | AAGGATTTATCAGAAATTTTGATATCCCTGCACGCCAAGTCATTCAAAGCAAGATAATAGGAGATCCACAGCAGATGCAGTGAACAAGCCTTCCGGCTTACCTGCTGGCCCTGAAACACAT | AAGCATAATAGGAGAACCAC |
| TDA3 | TATGCACCCACCGTTTCAAACGTACCCTTCAAAGAACAGCGAAGGGAAATAACATATAGTCAGTTAGGTGGCTTAATCATCGGTTGCTGTACTGCTTACTACTTGACCCAGCATCCAAGTTTCA | CATATTGTCATTGTAGGTGG |
| VPS5 | TCAATTGGTCAAATATCTACAAATGAAGAAAGAAAAGACAATGAGCTTTAAGAGACAACAGCCTCATAAACAAGATAGACTTGGATAGTGCGCCAGAATGGAAAGACCCTGGTTTGTCG | AGAGACCACAGCTTCATTTG |

## Table S4. Plasmids used in this study

| ID | Plasmid Structure | Source |
|---|---|---|
| MT4954 | pKS1ST | DualSystems Biotech |
| MT4651 | pD1204-GAL1-MATaWT-[CDTb-B12-Light chain, Fab']-FLAG-TCyc1-URA3 | This study |
| MT4652 | pD1204-GAL1-MATaAla22-[CDTb-B12-Light chain, Fab']-FLAG-TCyc1-URA3 | This study |
| MT4653 | pD1204-GAL1-MATaVal22-[CDTb-B12-Light chain, Fab']-FLAG-TCyc1-URA3 | This study |
| MT4654 | pD1204-GAL1-BBaK-[CDTb-B12-Light chain, Fab']-FLAG-TCyc1-URA3 | This study |
| MT4655 | pD1204-GAL1-Lysozyme-[CDTb-B12-Light chain, Fab']-FLAG-TCyc1-URA3 | This study |

| | | |
|---|---|---|
| MT4656 | pD1204-GAL1-Invertase-[CDTb-B12-Light chain, Fab']-FLAG-TCyc1-URA3 | This study |
| MT4657 | pD1204-GAL1-Glucanase-[CDTb-B12-Light chain, Fab']-FLAG-TCyc1-URA3 | This study |
| MT4658 | pD1204-GAL1-Amylase-[CDTb-B12-Light chain, Fab']-FLAG-TCyc1-URA3 | This study |
| MT4659 | pD1204-GAL1-Inulase-[CDTb-B12-Light chain, Fab']-FLAG-TCyc1-URA3 | This study |
| MT4660 | pD1204-GAL1-MATaWT-scFvGC132-FLAG-TCyc1-URA3 | This study |
| MT4661 | pD1204-GAL1-MATaWT-LaG16 VHH-FLAG-TCyc1-URA3 | This study |
| MT4663 | pGZ110-Cas9-amdSYM | This study |
| MT4664 | pGZ110-Cas9-amdSYM-URA3sgRNA | This study |
| MT4665 | pKS-pADH2- MAT α WT -J3r-FLAG-TCyc1-KanMX | This study |
| MT4666 | pKS-pADH2- MAT α WT -L8Cj3-FLAG-TCyc1-KanMX | This study |
| MT4667 | pKS-pADH2- MAT α WT -L911F1F-FLAG-TCyc1-KanMX | This study |
| MT4668 | pKS-pADH2- MAT α WT -A4.2-FLAG-TCyc1-KanMX | This study |
| MT4669 | pKS-pADH2- MAT α WT -A20.1-FLAG-TCyc1-KanMX | This study |
| MT4670 | pKS-pADH2- MAT α WT -A26.8-FLAG-TCyc1-KanMX | This study |
| MT4671 | pKS-pADH2- MAT α WT -4NC2-FLAG-TCyc1-KanMX | This study |
| MT4672 | pKS-pADH2- MAT α WT -FlagV1-FLAG-TCyc1-KanMX | This study |
| MT4673 | pKS-pADH2- MAT α WT -FlagV6-FLAG-TCyc1-KanMX | This study |
| MT4674 | pKS-pADH2- MAT α WT -K922-FLAG-TCyc1-KanMX | This study |
| MT4675 | pKS-pADH2- MAT α WT -4PC2-FLAG-TCyc1-KanMX | This study |
| MT4676 | pKS-pADH2- MAT α WT -5M2I-FLAG-TCyc1-KanMX | This study |
| MT4677 | pKS-pADH2- MAT α WT -5M2J-FLAG-TCyc1-KanMX | This study |
| MT4678 | pKS-pADH2- MAT α WT -5M2M-FLAG-TCyc1-KanMX | This study |
| MT4681 | pKS-pADH2- MAT α WT -VH303-FLAG-TCyc1-KanMX | This study |
| MT4682 | pKS-pADH2- MAT α WT -VH-1bag-FLAG-TCyc1-KanMX | This study |
| MT4683 | pKS-pADH2- MAT α WT -5M2M-5M2I-FLAG-TCyc1-KanMX | This study |
| MT4684 | pKS-pADH2- MAT α WT -m3F-FLAG-TCyc1-KanMX | This study |
| MT4685 | pKS-pADH2- MAT α WT -m3F-6xHIS-TCyc1-KanMX | This study |
| MT4686 | pKS-pADH2- MAT α WT -m3F-m3F-FLAG-TCyc1-KanMX | This study |
| MT4687 | pKS-pADH5- MAT α WT -m3F-FLAG-TCyc1-KanMX | This study |
| MT4688 | pKS-pPGK1- MAT α WT -m3F-FLAG-TCyc1-KanMX | This study |
| MT4689 | pKS-pTDH3- MAT α WT -m3F-FLAG-TCyc1-KanMX | This study |
| MT4690 | pKS-pTEF1- MAT α WT -m3F-FLAG-TCyc1-KanMX | This study |
| MT4691 | pKS-pADH5- MAT α WT -m3F-m3F-FLAG-TCyc1-KanMX | This study |
| MT4692 | pKS-pPGK1- MAT α WT -m3F-m3F-FLAG-TCyc1-KanMX | This study |
| MT4693 | pKS-pTEF1- MAT α WT -m3F-m3F-FLAG-TCyc1-KanMX | This study |
| MT4697 | pKS-pGAL1- MAT α WT -5M2I-6xHIS-TCyc1-KanMX | This study |
| MT4698 | pKS-pGAL1- MAT α WT -5M2I-6xHIS-TCyc1-KanMX | This study |
| MT4699 | pKS-pGAL1- MAT α WT -5M2M-5M2I-6xHIS-TCyc1-KanMX | This study |
| MT4700 | pKS-ppADH2- MAT α WT -J3r-6xHIS-TCyc1-KanMX | This study |
| MT4701 | pKS-pADH2- MAT α WT -A5.1-FLAG-KanMX | This study |

Disease Models & Mechanisms • Supplementary information

**Table S5. Strains used in this study**

| ID | Yeast | Genotype | Origin |
|---|---|---|---|
| MTy5073 | *S. boulardii* | MYA-796 | ATCC |
| MTy1580 | *S. cerevisiae* | Sigma1278b | Tyers lab |
| MTy5084 | *S. boulardii* | MYA-796 *ura3* | This study |
| MTy5087 | *S. boulardii* | MYA-796 hda2 | This study |
| MTy5088 | *S. boulardii* | MYA-796 *tda3* | This study |
| MTy5089 | *S. boulardii* | MYA-796 *vps5* | This study |
| MTy5090 | *S. boulardii* | MYA-796 tda3 *vps5* | This study |
| MTy5091 | *S. boulardii* | MYA-796 *hda2 vps5 tda3* | This study |

**Table S6. Disease activity index score scale**

| Score | Weight loss | Stool consistency | Occult/gross bleeding |
|---|---|---|---|
| **0** | 0 | Normal | Normal |
| **1** | 1 – 5% | | |
| **2** | 6 – 10% | Loose | |
| **3** | 11 – 15% | | |
| **4** | >15% | Diarrhea | Black stool |

*Normal stool* well-formed pellet; *loose stool*-pasty stools that do not stick to the anus; *diarrhea*-liquid stools that stick to the anus and/or surrounding area.

Disease Models & Mechanisms • Supplementary information
